# Baseline of Physiological Body Temperature and Hematological Parameters in Captive *Rousettus aegyptiacus* and *Eidolon helvum* Fruit Bats

**DOI:** 10.3389/fphys.2022.910157

**Published:** 2022-08-29

**Authors:** Melanie Rissmann, Virginia Friedrichs, Nils Kley, Martin Straube, Balal Sadeghi, Anne Balkema-Buschmann

**Affiliations:** ^1^ Institute of Novel and Emerging Infectious Diseases, Friedrich-Loeffler-Institut, Greifswald, Germany; ^2^ Department of Viroscience, Erasmus MC, Rotterdam, Netherlands; ^3^ Institute of Immunology, Friedrich-Loeffler-Institut, Greifswald, Germany; ^4^ Landratsamt Ortenaukreis, Amt für Veterinärwesen und Lebensmittelüberwachung, Offenburg, Germany

**Keywords:** fruit bat, *Rousettus aegyptiacus*, *Eidolon helvum*, exotic animal husbandry, physiology, hematology, clinical chemistry

## Abstract

The discovery of bats as reservoir hosts for a number of highly pathogenic zoonotic agents has led to an increasing interest of infectious disease research in experimental studies with bats. Therefore, we established breeding colonies of *Rousettus aegyptiacus* and *Eidolon helvum* fruit bats, which both have been identified as reservoir hosts for relevant zoonotic disease agents, such as Marburg virus and Lagos bat virus. Since 2013, individuals of both species have been recruited to the Friedrich-Loeffler-Institut (FLI) from zoological gardens in Europe, to where these species had been introduced from the wild several decades ago. The aviaries have been designed according to national recommendations published by the Federal Ministry of Agriculture. Under these conditions, both species have been reproducing for years. To better understand the physiology of these animals, and to generate baseline knowledge for infection experiments, we monitored the body core temperatures of *R. aegyptiacus* bats in the aviaries, and found a circadian variation between 34°C and 41.5°C. We also determined the hematological parameters of both species, and detected specific differences between both bat species. For values of clinical chemistry, no correlation to age or sex was observed. However, species-specific differences were detected since ALT, BUN and CREA were found to be significantly higher in *R. aegyptiacus* and GLU and TP were significantly higher in *E. helvum* bats. A higher hematocrit, hemoglobin and red blood cell level was observed in subadult *R. aegyptiacus*, with hemoglobin and red blood cells also being significantly increased compared to *E. helvum*. Lymphocytes were found to be the dominant white blood cells in both species and are higher in female *E. helvum*. Neutrophil granulocytes were significantly higher in *E. helvum* bats. This underlines the necessity to define baseline profiles for each bat species prior to their use in experimental challenge.

## Introduction

Pteropodid bats have recently been identified as reservoir hosts for a number of infectious disease agents exhibiting high zoonotic potential, which can cause severe disease in humans and/or livestock animals (Han et al., 2015; Moratelli and Calisher, 2015). Due to their unique physiology, bats may propagate and disseminate disease agents at higher levels and over longer periods of time than most other mammals: Many bat species live in colonies that represent the largest intraspecific cumulation among mammals, thus facilitating intraspecies transmission. Moreover, given their ability to fly (Calisher et al., 2006; O'Shea et al., 2014), to migrate over large distances of up to several thousand kilometers and their global distribution sparing only polar regions, bats have the prerequisite to efficiently spread infectious agents over large distances. Their body temperature may physiologically vary between below 20°C during torpor or hibernation, and above 41°C during flight, upon which the metabolism of a bat increases up to 34-fold (Thomas and Suthers, 1972; George et al., 2011; Hayman, 2016). This variability in body temperature may have facilitated the evolutionary adaption of bat-borne viruses to varying host body temperatures, thereby contributing to a high viral pathogenicity during a hyper- or hypothermic status of other mammals (O'Shea et al., 2014).

In recent years, the increasing overlap between human and wildlife habitats, caused by rapid progression of human invasion into numerous ecosystems, intensified agriculture, as well as ecological and climatic changes have resulted in considerably closer contact between humans, livestock, and wildlife such as bats, therefore leading to an increased risk of interspecies disease transmission (O'Shea et al., 2014; Plowright et al., 2015). As a result, many viruses which may have been circulating unnoticed in wildlife for many generations, were only recently discovered as pathogens affecting humans after unprecedented spillover events.

Although bats and their unique immunological traits have gained increasing scientific attention in recent years, a number of immunological mechanisms underlying their exclusive role as viral reservoirs remain enigmatic to date. Recent work has uncovered fundamental differences in immunity between bats and other species: Bats have the ability to efficiently respond to RNA virus infection since interferon (IFN) is constitutively expressed in steady state (Bondet et al., 2021). Moreover, the variation in body core temperatures may restrict viral replication, especially since the increased temperature during flight supports constitutive *IFNA* expression (Bondet et al., 2021; Fumagalli et al., 2021). This temperature-dependent expression of IFNs possibly leads to refined immune evasion strategies of bat-borne viruses, thereby increasing their pathogenicity in other species (Irving et al., 2021). Furthermore, sensing of viral DNA is dampened, as indicated by restrained inflammasome formation and function (Ahn et al., 2016; Xie et al., 2018; Ahn et al., 2019; Subudhi et al., 2019).It could be shown that this reduced inflammasome activity is a result of the unique chiropteran physiology, leading to constitutive activation of the heat shock response (HSR) (Chionh et al., 2019). As self-powered flight is an energetically demanding way of locomotion, associated with immense heat production by the wing muscles (Powers et al., 2015; Powers et al., 2017; Rummel et al., 2019), bats evolved towards elevated HSR induction. This is analogous to birds, but even more pronounced in bats, and subsequently leads to unparalleled viability of bat cells in high temperatures compared to their avian counterparts (Chionh et al., 2019). Probably the most striking fact is the general propensity of bats to harbor a plethora of highly pathogenic viruses while hardly displaying any clinical signs of disease. Zoonotic viruses originating from bats include lyssaviruses (Johnson et al., 2010), Ebola and Marburg viruses (Swanepoel et al., 2007), Hendra and Nipah viruses (Halpin et al., 2000), coronaviruses such as MERS-CoV and possibly SARS-CoV-2 (Zhou et al., 2020), and new viruses that are incessantly isolated from various species (Thomas and Suthers, 1972; Field et al., 2001; Calisher et al., 2006; Wang and Anderson, 2019). This viral richness indicates a long history of co-evolution between bats and bat-borne viruses (O'Shea et al., 2014). More specifically, the straw-colored fruit bat *Eidolon helvum* has been shown to carry lyssaviruses, esp. Lagos bat virus (LBV (Suu-Ire et al., 2017)), paramyxoviruses including henipaviruses (Hayman et al., 2008; Baker et al., 2012), flaviviruses, esp. Zika virus (Fagre et al., 2021), as well as adenoviruses (Ogawa et al., 2017). Antibodies against Ebola virus (Hayman et al., 2010; Hayman et al., 2012) and Crimean-Congo-hemorrhagic fever virus (Muller et al., 2016) have also been detected in this species. The Egyptian rousette bat, *Rousettus aegyptiacus,* has been identified as a reservoir of the marburgviruses, MARV and RAVN virus (RAVV), and other filoviruses (Towner et al., 2009; Amman et al., 2012; Paweska et al., 2012; Schuh et al., 2017), poxviruses (David et al., 2020), as well as paramyxoviruses such as Sosuga virus (Amman et al., 2015; Markotter et al., 2019; Amman et al., 2020). Interestingly, a role as natural reservoir of Ebola virus (EBOV) is unlikely for *R. aegyptiacus*, since experimental infection did not result in virus shedding (Jones et al., 2015; Paweska et al., 2016). For other viruses such as henipaviruses, a reservoir role has been assumed (Markotter et al., 2019), but further studies are needed to identify specific reservoir host-virus relationship. Therefore, both species are highly relevant for infectiology and zoonoses research, since gaining knowledge concerning the mechanisms of inter- and intraspecies transmission, immune response and pathogenic processes are essential for our understanding and risk perception. Husbandry and breeding of both bat species under defined hygiene conditions is highly relevant to prospectively address questions regarding their specific role in the replication and transmission of potentially zoonotic disease agents. However, only very few research institutions have the required facilities and conditions to house fruit bat breeding colonies in a species-appropriate manner. Therefore, the husbandry of *Rousettus* and *Eidolon* breeding colonies at the FLI in indoor aviaries, as well as the maintenance of these animals in specialized cages under experimental conditions in the biosafety level (BSL)-2 to BSL-4 facilities pose an exceptional infrastructure and are therefore described in this paper.

In recent years, a number of experimental challenge studies have been performed on these two species. For example, an experimental challenge of *E. helvum* with LBV revealed that these animals develop clinical disease associated with meningoencephalitis after intramuscular challenge with a median infectious dose of 10^2^ TCID_50_ (tissue culture infectious dose) (Begeman et al., 2020). Meanwhile, *R. aegyptiacus* bats have already been experimentally challenged with a number of different zoonotic viruses. Experimental infection with Nipah virus (NiV) demonstrated that this bat species does not support NiV replication (Seifert et al., 2020) which suggests that this bat species does not play a role in the transmission of henipaviruses in Africa. An experimental challenge of *R. aegyptiacus* bats with MARV confirmed the susceptibility of this species to infection with this virus, developing a very mild clinical disease upon challenge (Jones et al., 2019). In contrast, these bats were shown to be refractory to an infection with Ebola virus (Paweska et al., 2016). While these animals also seemed resistant to an infection with SARS-CoV (van Doremalen et al., 2018), a challenge of this species with SARS-CoV-2 resulted in virus propagation and shedding of low titers within the first 6 days (Schlottau et al., 2020). Experimental challenge with Sosuga virus, a human pathogenic paramyxovirus isolated from *Rousettus* bats in Uganda after transmission to a human patient (Amman et al., 2015), resulted in subclinical infection in these bats, supporting the hypothesis that this species may play a role as reservoir of this virus (Amman et al., 2020). Challenge of this species with the Rift Valley Fever Virus (RVFV) vaccine strain MP12 as well as the more virulent strain 35/74 confirmed this bat species’ general susceptibility to an infection with this virus, developing a subclinical infection and a short and limited shedding of virus within the first few days after challenge (Balkema-Buschmann et al., 2018) (Rissmann et al., unpublished data). Finally, experimental challenge of this species with a bat influenza H9N2 strain isolated from this species also resulted in a subclinical infection with a short period of virus shedding (Halwe et al., 2021). These studies confirm the general susceptibility of both bat species to infections with human pathogenic viruses, without causing clinical disease.

Both fruit bat species of interest for this manuscript feed on a variety of different fruits, supplemented by nectar and pollen from a variety of flowers. Occasionally, buds and leaves are also chewed and their juice is consumed. The ingestion of tree sap (*Eidolon*) and insects (*Rousettus*) has also been observed (Wilson et al., 2019). Both species can live up to over 20 years of age in captivity (Kwiecinski and Griffiths, 1999; Wilkinson and South, 2002; Weigl et al., 2005), and both species are polygynandrous. In general, *Rousettus* have two annual breeding seasons. After a gestation period of 115–120 days, one, rarely two pups are born. The pups become fully independent of their mothers after 9 months, but tend to stay within the colony their whole lives (Okia, 1987). The main breeding season of *Eidolon* occurs from April until June. In some populations, an embryonic diapause has been observed that lasts until October, followed by the birth of the usually single pup between February and March, to coincide with rainy and thus main fruit seasons. In other populations, the pups are born 4 months post mating, without any delay (Kingdon, 1984).

The distribution of both known species of the genus *Eidolon* is restricted to Africa. *E. helvum* is a medium-sized fruit bat species with the mean measurements of head-body: 150–195 mm, forearm 117–132 mm, and a mean body weight of 230–350 g. Females are slightly smaller than males (Wilson et al., 2019). *E. helvum* is widespread in most regions of Sub-Saharan Africa (Solari and Baker, 2007). However, in Madagascar, *Eidolon dupreanum* occurs (Wilson et al., 2019). In many parts of its distribution area, *E. helvum* is a nomadic species, especially during non-breeding season (Cotterill, 2001). Depending on the seasonal availability of food, they may travel over up to 100 km per night (Fahr et al., 2015). While the majority of the population may be non-nomadic, some individuals will travel thousands of kilometers, following the local fruit and flower seasons. Another study using radio tracking of individual bats even revealed one individual moving 370 km in one night, while another individual travelled a cumulative 2,518 km in 140 days (Richter and Cumming, 2008). Interestingly, a genetic exchange seems to occur among colonies within the entire distribution range. Tropical forests are their main habitat where they roost in groups, with migratory movements into savannas, gallery and swamp forests up to 2,000 m above sea level (Wilson et al., 2019). Colony sizes usually range around 100 individuals, but locally and seasonally can comprise up to 8,000,000 individuals, which is one of the largest localized migration accumulation of individual specimens among non-human mammals (Sørensen and Halberg, 2001; Racey, 2004; Richter and Cumming, 2008). Roosts are mainly located in trees, where *Eidolon* bats roost freely on branches and trunks at heights of 6–20 m above ground in direct physical contact with conspecifics. Entrance areas of caves and rocks are also locally used as roosts. *Eidolon* colonies are regularly found roosting in proximity of human settlements, occasionally even in city centers. Eidolon bats do not echolocate. This fruit bats navigate mainly with the help of their very efficient eyesight and a well-developed sense of smell (Wilson et al., 2019). Eidolon bats forage at average distances of up to 59 km from their roost (Richter and Cumming, 2008).

The seven species of the genus *Rousettus* are mainly found in tropical Asia. *R. aegyptiacus* is the only representative species of its genus on the African mainland. It is a small to medium sized fruit bat, with head-body measurement of 138–192 mm, forearm 82–106 mm, and a median body weight of 81–171 g. Females are smaller than males. These fruit bats are abundant in a number of separated populations in Sub-Saharan Africa. They are also found in the Nile Valley, on Cyprus, along the south coast of Turkey and on the Arabian Peninsula (Solari and Baker, 2007). It is the most distant fruit bat species from the equator and with the Cypriot population, the only Pteropodid bat species endemic to Europe. *R. aegyptiacus* bats mainly inhabit underground spaces such as caves, mines and tunnels, as well as cellars and temples. Their distribution is influenced more by the availability of suitable roosting sites than vegetation associations. At least seasonally, individuals and small colonies also roost in trees with dense branches, especially palm trees. Inside caves they cluster in dark corners and crevices both on the ceiling and on the walls down to just above the ground. Their habitat ranges from tropical forests to arid regions. Especially populations in dry regions often live in the immediate vicinity or in the middle of human settlements, following the greater availability of food, water and roosts. The animals form colonies that can include approximately 100 to many thousands (40,000 to 50,000) individuals (Wilson et al., 2019; Monadjem et al., 2020). While *R. aegyptiacus* bats show no extended migration behavior, many populations show seasonal migration following fruit abundance (some up to 500 km). Exchange of individuals between roosts occurs regularly (Jacobsen and Du Plessis, 1976; Wilson et al., 2019). These fruit bats spend the day in direct physical contact with conspecifics and regularly share their roosts with a number of other bat species. *R. aegyptiacus* bats use echolocation in their very dark roosts. In the field they navigate mainly using their very efficient eyesight and a good sense of smell (Wilson et al., 2019). Radio-tracked individuals were shown to fly about 24–25.5 km from their roosting cave to feeding sites, a journey that took 90 min (Jacobsen and Ferguson, 1985; Tsoar et al., 2011).

Since apparent clinical manifestations after viral infections are rarely observed in bats, the analysis of hematological and plasma biochemical values can be a useful tool to elucidate pathomechanisms and correlates of infection. Extensive field studies already demonstrated that viral infections of bats can be associated to alterations in the hematology and clinical chemistry (McMichael et al., 2015; McMichael et al., 2019), which highlights the added value of those analysis for future experimental setups. The composition of the blood and according biomarkers is affected by various intrinsic factors such as age and gender, as well as by external factors such as the season, time of day, food availability and quality etc. (Westhuizen, 1978; Minematsu et al., 1995; McMichael et al., 2015; McMichael et al., 2019). As described previously, most reliable data are therefore generated from populations that are consistently kept at the same diet and husbandry for a prolonged period to avoid impairment of variable extrinsic factors (Selig et al., 2016). Age-dependent variations of hematological and biochemical parameters were observed in diverse bat species, with elevated levels of alkaline phosphatase and decreased levels of hemoglobin, hematocrit, as well as white and red blood cells in pre-mature animals being the most consistent findings (Hall et al., 2014; McMichael et al., 2015; Edson et al., 2018; Olopade et al., 2020; Moretti et al., 2021). Additionally, although to a variable extent, different studies also reported sex-dependent characteristics such as higher white blood cells in females and higher alanine aminotransferase (ALT) values in males (McLaughlin et al., 2007; Hall et al., 2014; McMichael et al., 2015; Edson et al., 2018). For the analysis and interpretation of data from hematology and clinical chemistry, it is of utmost importance to be aware of both age-, sex- and species-dependent variations. Therefore, we determined the influence of these intrinsic factors by collecting blood from female and male, as well as adult (>2 years) and subadult *R. aegyptiacus* and *E. helvum* bats. Due to the limited number of captive breeding colonies of both species in research facilities worldwide, almost no data is available concerning the physiological parameters of these animals regarding body temperature, hematology, blood chemistry and immunology (see references in Table 2). Importantly, to date, only limited reports of clinical reference levels have been published. Blood from both healthy control and infected animals is routinely analyzed during viral pathogenesis studies, but determining significant hallmarks of the disease is very challenging without steady state values as a reference database for comparison purposes. Physiological baselines will provide better insight into the overall health of fruit bats and validate deviations from expected ranges as clinically relevant when they are used in experimental setups. We therefore established reference levels for body core temperature, hematology, clinical chemistry in these two healthy fruit bat populations.

## Materials and Methods

### Establishment of Fruit Bat Breeding Colonies at the FLI

Individuals of both fruit bat species were retrieved from zoological gardens in Europe. While most of these animals were adult and had already been reproductive, some sub-adult animals were also obtained. Prior to their transfer, the selected individuals were thoroughly examined, and swab samples were collected for an initial virological screening. No indication of infections with relevant pathogens (coronaviruses, paramyxoviruses, lyssaviruses, as well as bacterial pathogens that would have contradicted the introduction into the small animal breeding unit) were detected. Upon transfer, the animals were again monitored through repeated oral swab sampling, collection of fecal and urine samples, as well as through analysis of sentinel mice (C57Bl/6 and IFNAR−/−) that were kept for at least 2 weeks in close contact with the bats. The bat samples as well as tissues of the sentinel mice were analyzed for the above-mentioned agents. Again, no viral disease agents of concern were detectable in the colonies. However, the samples collected in the *E. helvum* animal room revealed a subclinical infection with *Klebsiella* and *Citrobacter* spp. All animals were individually tagged with a subcutaneous transponder (Virbac backhome, Bad Oldesloe, Germany) between the shoulder blades. For reading the ID in this system, the reader must be held in a maximal distance of 10–15 cm from the animal, which is in most cases only possible when the animal is caught.

### Husbandry

**FIGURE 1:**
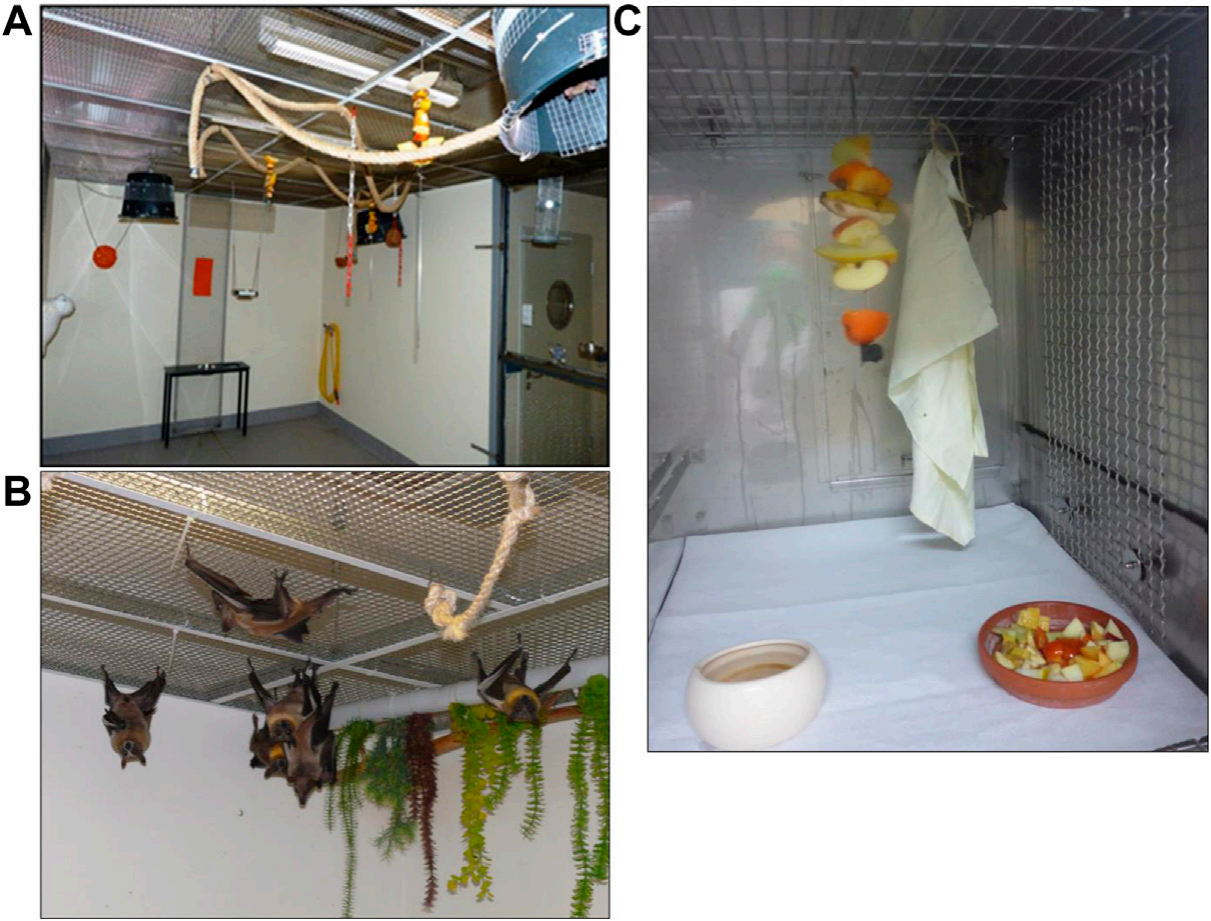
Housing and husbandry in the aviary **(A)** *R. aegyptiacus,* **(B)** *E. helvum*, and **(C)** *R. aegyptiacus* in a cage during an infection study. The aviary offers several possibilities to climb, fly and hide, allowing bats to show their natural behavior. During infection studies, caged animals can climb and hide, and can even minimally fly. Social interactions are possible in both settings. In both settings, the daylight cycle was from 6:00 to 18:00, with a 15 min dimming phase between day and night cycles.

The breeding colonies are kept in aviaries spanning 22 m^2^ lined with a metal mesh ceiling with a mesh size of 1.5 cm at 2.30–2.50 m height, allowing the animals to hang from the ceiling in their physiological posture, in groups of up to 20 individuals. In the *R. aegyptiacus* aviaries, two to three bins with a diameter of 20–50 cm and coated with a fine metal mesh are positioned upside down from the ceiling as a retreat location for the animals. When entering the room, at least half of the animals are found in these buckets, showing the high acceptance of these hiding areas. In the corners of the room, plastic plants of 20 cm length hang from the ceiling at approx. 15 cm distance to the walls, again offering a hiding area. Two long ropes of 4 cm diameter cross the room at different heights to offer additional hanging and climbing options, especially during feeding (Figure 1A). This still offers the animals which are extremely skillful in maneuvering around obstacles, sufficient free space for flying in the aviary. Although the maintenance of these colonies in captivity was conducted for >50 years, *Rousettus* individuals are very shy and will in most cases not allow a human to approach closer than 50–70 cm. The *E. helvum* aviary (Figure 1B) is equipped differently, since these animals do not use the bins as a retreat area. Here, jute bags are attached to the ceiling in all corners of the room at a distance of 20–25 cm. More of the 4 cm rope is hanging from the ceiling, since these animals rarely fly, giving them sufficient opportunities to climb. These animals are less shy, and some individuals will accept handfeeding with favored fruits such as banana or melon.

At regular intervals, all animals are caught and inspected thoroughly for any possible injuries or other anomalies which might remain unnoticed without handling the animals individually. At that occasion, all offspring are tagged with an individual transponder (Virbac Backhome) for identification. During infection studies, up to three (*Eidolon*) or four (*Rousettus*) individuals are kept in stainless steel double cages (L x W x H 133 cm × 66 cm x 78 cm). A metal grid is mounted at the ceiling of the cages, allowing the animals to hang upside down (Figure 1C). A partition can be inserted for cleaning and during manipulation of the animals. Manipulations such as inoculation or blood sampling are carried out upon short isoflurane anesthesia. Bite-resistant gloves are worn when catching and manipulating the animals. Both species require similar climatic and nutritional conditions, i.e. room temperatures of 24–26°C and a humidity of >60%. The humidity level is maintained by rinsing the walls and floor daily during maintenance. The daylight cycle is from 6:00 to 18:00, with a 15 min dimming phase between day and night cycles. The diet consists of varying fruits and vegetables, with the avoidance of citrus fruits to prevent excessive vitamin C uptake. Since the commercially sold fruits contain high sugar levels, it is made sure that vegetables are also offered daily. Besides offering small pieces of <2 cm size in a bowl, the animals are also offered half or quartered unpeeled fruits in order to ensure sufficient activity of the animals during feeding.

### Determination of Body Temperature and Circadian Activity in *R. aegyptiacus* Bats

In order to analyze the physiological oscillation of the body temperature in *R. aegyptiacus*, temperature transponders (Anipill transponders, BodyCap, France) were implanted in four individuals, which regularly transmitted the temperature to a receiver that was mounted in proximity of the animals. In this experiment, we implanted transponders subcutaneously between the shoulder blades, or intraperitoneally in two individuals each. These individuals were observed in the aviary for fluctuations in body temperature, and measurements were taken every 2 minutes for up to 57 days. We only included days where measurements were at least 2/3 complete, resulting in 17 individual measuring days for this group. Measurements in cages were obtained from six individuals, with measurements every 5 minutes over a period of up to 12 days (theoretically resulting in 72 individual daily measurements), out of which 29 days with at least 2/3 complete readings were included in the analysis. The subcutaneous implantation, as a less severe manipulation of the animal, which would for animal welfare aspects be the favorable method, will measure a slightly lower temperature than the body core temperature. The intraperitoneal implantation, requiring a more severe manipulation, will measure the actual body core temperature. In all cases, the implantation was performed under isoflurane anesthesia with an application of Meloxicam (Metacam^®^; Boehringer Ingelheim, Ingelheim am Rhein, Germany) 1 h prior to the surgery. Days on which the transponders were implanted and the first day of acclimatization of animals transferred into a cage were excluded from analysis to minimalize stress-related variations. Due to the low number of *E. helvum* bats in our colony, this study involving invasive implantation of temperature transponders was only feasible in *R. aegyptiacus* bats.

To assess the circadian level of activity, a wildlife camera with automated activation upon locomotion activity was mounted in the aviary. The quantity of camera activation was used to determine the time zones of minimal and maximal activity. The activation times of an exemplary phase of 5 days was used for an initial estimation of the circadian activity.

### Hematology and Clinical Chemistry

To obtain reference values for biochemical and hematological values of *R. aegyptiacus* and *E. helvum*, a total of 60 animals of both sexes and different ages were tested (Table 1). All sampling procedures are performed during the morning, generally between 9:00 and 11:00. This is also the time when maintenance activities (cleaning and feeding) are performed, so the animals are used to being disturbed at this time of the day. Blood was collected from the lateral wing vein of each anesthetized bat (2–5% isoflurane anesthesia) by using a 27-gauge needle. Collected blood was immediately transferred into serum (10 *R. aegyptiacus* bats) or EDTA microtainer tubes (Sarstedt, Nümbrecht, Germany). Within a maximum of 3 hours after collection, blood was analyzed using the horse profile in a VetScan HM5 hematology analyzer (Abaxis, Union City, CA). The following hematological parameters were determined, with a full statistical analysis for the values determined for the parameters marked with an asterisk: white blood cell count (WBC*); neutrophil (NE*), lymphocyte (LY*), monocyte (MON), eosinophil (EO), and basophil (BA) percentages; red blood cell count (RBC*); hemoglobin (HGB*); hematocrit (HCT*); mean corpuscular volume (MCV); mean corpuscular hemoglobin (MCH); mean corpuscular hemoglobin concentration (MCHC); platelet count (PLT); plateletcrit (PCT), red cell distribution width (RDW); platelet distribution width (PDW) and mean platelet volume (MPV). After hematological analysis, whole blood was centrifuged (4,000 rpm, 10 min, 4°C) and plasma was stored at -20°C until further processing. All plasma samples as well as additional serum samples were tested on the VetScan VS.2 clinical chemistry analyzer (Abaxis) with the prep profile II, including alanine aminotransferase (ALT)*, alkaline phosphatase (ALP*), creatinine (CREA*), glucose (GLU*), total protein (TP*), and blood urea nitrogen (BUN*). For ten of the *Rousettus* bat samples, only the blood chemistry profile could be analyzed.

**TABLE 1:** Number of animals included in the study.

	n		
*Rousettus aegyptiacus*	40^a^		
			n
male	8	*♂ adult*	3
		*♂ subadult*	5
female	32	*♀ adult*	12
		*♀ subadult*	19
adult	15		
subadult^b^	25		
	n		
*Eidolon helvum*	20		
			n
male	13	*♂ adult*	10
		*♂ subadult*	3
female	7	*♀ adult*	5
		*♀ subadult*	2
adult	15		
subadult^b^	5		

a 10 animals were only analysed in blood chemistry.

b younger than 2 years.

### Statistical Analysis

The body temperature of four individuals was continuously measured for 17 days in the aviary, and for six ainmals in the cages for 12 days. A 24-h interval for each animal was selected randomly for data analysis, to avoid any bias of multiple time points for each sample. Outliers were removed using the Smirnoff-Grubbs rejection test and for evaluating the normal distribution we used the D'Agostino-Pearson test. Data were analyzed using Prism (version 7.01, GraphPad Software, La Jolla, CA, United States). Unpaired *t*-test was performed to assess the statistical significance of the determined clinical blood chemistry (ALT, BUN, TP, ALP, CREA, GLU; *R.aegyptiacus*, n = 40; *E.helvum*, *n* = 20) and hematology (HCT, HGB, LY, NE, RBC, WBC; *R.aegyptiacus*, *n* = 30; *E.helvum*, *n* = 20) values between both species. Statistical analysis was performed using SPSS software (IBM Corp. Released 2011. IBM SPSS Statistics for Windows, Version 20.0, IBM Corporation, Armonk, NY, United States). *p*-value < 0.05 was considered statistically significant.

To assess a possible correlation between the clinical chemistry and hematology values and sex and age of the individuals, Spearman’s correlation test was applied (analysis was performed using SPSS software) with a significance level set at *p*-value < 0.05.

## Results

### Body Core Temperature and Circadian Activity of Captive *R. aegyptiacus* Bats

**FIGURE 2:**
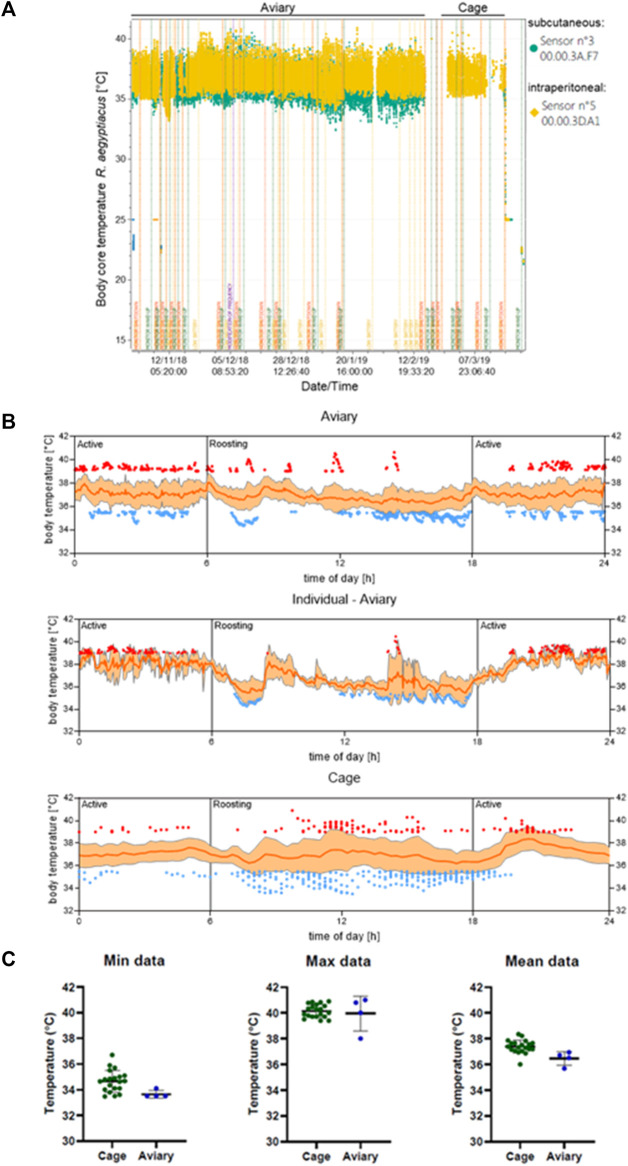
Body core temperature of *R. aegyptiacus* bats is dependent on the transmitter location. **(A)** The difference in determined body temperature of *R. aegyptiacus* bats is exemplarily shown for one bat with a subcutaneously implanted transponder (sensor n° 3), and one bat with an intraperitoneally implanted transponder (sensor n° 5, yellow). **(B)** The body temperature of individuals in the aviary (*n* = 4, a total of 17 individual measuring days with 11,495 values are depicted) ad in a cage (*n* = 6, a total of 29 individual measuring days with 2,735 values are depicted) was determined. Low (≤35.5°C) and high temperatures (≥39°C) of all individuals are indicated in blue and red, respectively. One individual carrying an intraperitoneal transmitter is displayed separately for clarity purposes. Data represents several days of measurement of all individuals carrying a transmitter. Orange line represents the mean, ±SD displayed as shading. **(C)** Mean as well as maximum and minimum values of animals carrying a intraperitoneal transmitter are displayed. The temperature was assessed from October-February (aviary) and February-March (cage) and values are depicted from 12:00 to 24:00.

In the aviary, a body core temperature minimum of 32.5°C and a maximum of 41°C were measured from the intraperitoneally implanted transponders, while a minimum of 32°C and a maximum of 41.5°C were determined by the subcutaneously implanted transponders (Figure 2A). After animals were transferred into cages and were allowed an acclimatization phase of 7 days, the body core temperature measured by the intraperitoneal transponders reached a maximum of 40.5°C and a minimum of 35.5°C. On average the temperature was higher when animals are kept in the cages (Figure 2).

**FIGURE 3:**
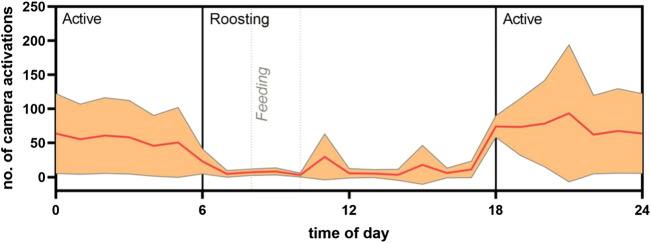
Proxy of circadian activity from frequency of camera activation. The mean number of camera activations in an aviary of 30 individuals of *R. aegyptiacus* bats was analyzed in association to the time of day. Camera activation is displayed in relation to active/roosting phases of the bats. Light regime: 6:00 lights on; 18:00 lights off (both with dimming effect). Orange line represents the mean, ±SD displayed as shading.

Overall, the body temperature pattern correlated with activity and roosting phases of the animals, with lower temperatures (≤35.5°C) prevailing during roosting, and higher temperatures (≥39°C) predominantly found during active phases. Strikingly, although the cages were too small to allow active flight, the body temperature of the caged animals increased during the active phase (Figure 2B). However, the oscillation range was considerably smaller in caged animals and the mean temperature was higher in animals kept in a cage. Comparative analyses revealed that this variation results from caged animals failing to reach low body core temperatures, e.g. 32.5°C like animals roaming the aviary (Figure 2C). Furthermore, analyses of the body core temperature pattern of one single individual in the aviary highlights the correlation between temperature increase and decrease in accordance with the circadian rhythm (Figure 2B, Figure 3). This coherence could also be detected for all observed animals in the aviary, although not as pronounced as for each individual. Once the aviary was illuminated (6:00 h), the colony engaged in roosting, which was accompanied by lower temperatures (Figure 2B) and less activity (Figure 3). It can be assumed, that body core temperature and activity increase during the roosting phases are associated with husbandry. In addition, increase of activity and increase of body core temperatures correlated with lights being switched off in the aviary (18:00 h, Figure 2, Figure 3).

Analysis of the circadian activity as assessed by the automatic activation of a wildlife camera that was mounted in the aviary on five consecutive days revealed a clear increase in activity during the night, with the highest activity in the first 4 hours of darkness in the aviary (Figure 3).

### Clinical Chemistry

**FIGURE 4:**
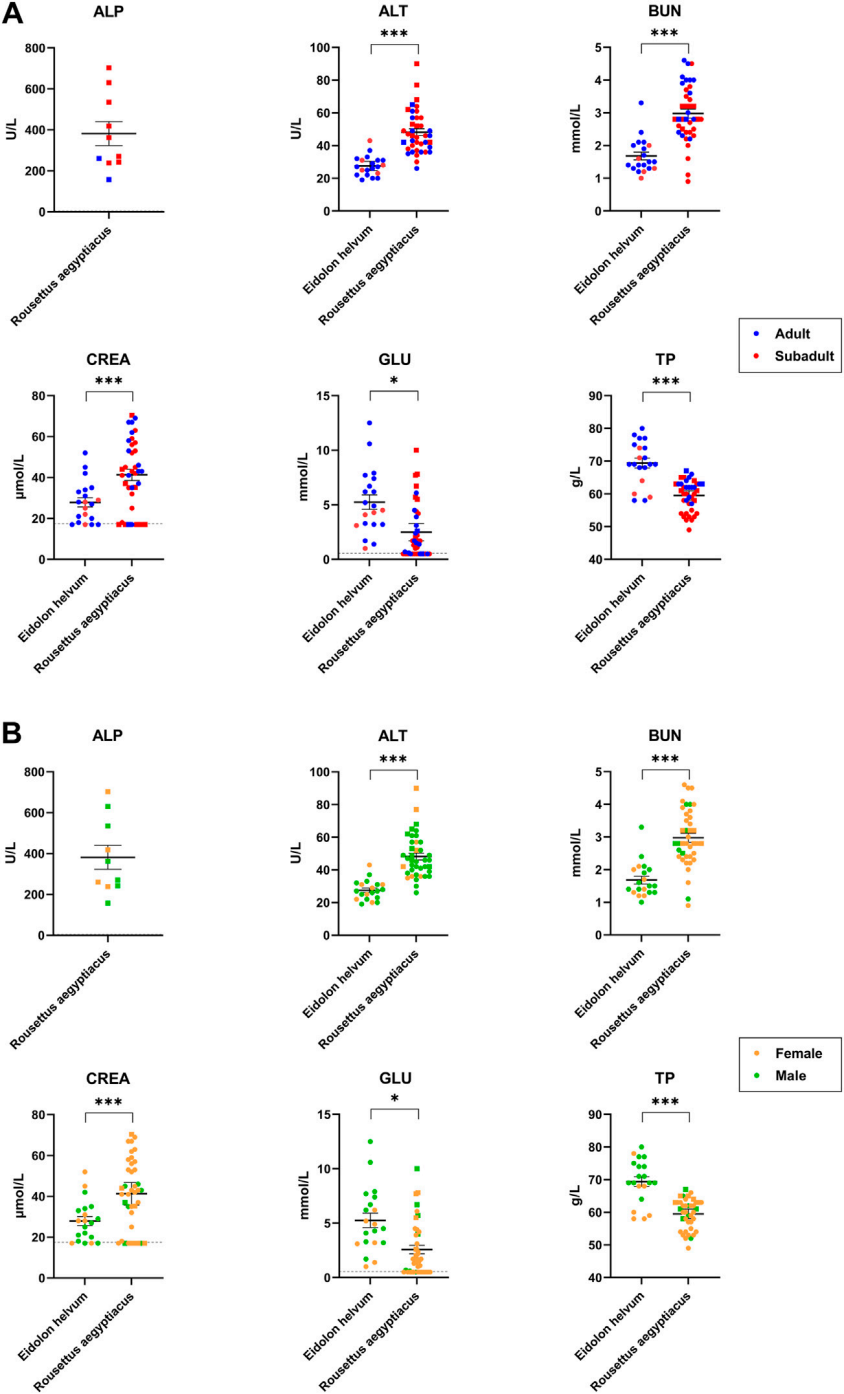
Age- and sex-dependent variations of clinical blood chemistry parameters in *R. aegyptiacus* and *E. helvum.* Single dots represent one animal (adult/subadult) in **(A)** and (female/male) in **(B)**. The same cohort was used for comparison. Dotted lines represent the lower detection limit. Solid horizontal bars represent mean ± SEM. Note that ALP could only be determined for 10 *R. aegyptiacus* bats. Statistically significant differences were determined for clinical blood chemistry parameters (ALT, BUN, TP, ALP, CREA, GLU), * = significant: *p*-value < 0.05; *** = extremely or highly significant: *p*-value < 0.001.

The six parameters ALT, ALP, CREA, GLU, TP, and BUN were determined to establish baseline values for the two species *R. aegyptiacus* and *E. helvum*, to detect inter-species differences (Supplementary Table S1), as well as to assess potential correlations between clinical blood chemistry values and age or sex of the individuals (Figure 4). Furthermore, detected values were compared to those previously reported for *R. aegyptiacus* and *E. helvum*, as well as other pteropodid species (Table 2). While no correlation to age or sex was observed for any of the investigated analytes, significant differences were detected for all parameters between the two bat species.

**TABLE 2:** Comparative values of blood chemistry parameters determined for pteropodid bat species

				Parameter							
				ALP (*U/L*)		ALT (*U/L*)	BUN (*mmol/L*)	CREA (*µmol/L*)	TP (*g/dl*)	GLU (*mg/dl*)	
This paper	*E. helvum*			-		26.40 (±6.64)	1.62 (±0.59)	29.23 (±10.51)	71.06 (±6.52)	5.43 (±2.58)	
	*R. aegypt.*			381.5 (±185.00)		48.20 (±13.14)	2.97 (±0.86)	47.53 (±13.14)	59.52 (±4.58)	3.45 (±2.53)	
Selig et al. (2016)	*E. helvum*			35 (±9.2)		46.2 (±27.4)	3.04 (±1.32)	25.52 (±8.8)	73.8 (±6.2)	6.33 (±1.85)	
Cavaleros et al. (2003)	*R. aegypt.*			wild: 576 (±130)	captive: 783 (±127)						
Westhuizen, (1978)	*R. aegypt.*									6.46 (±1.66)	
Moretti et al. (2021)	*R. aegypt.*			523 (±194)		46 (±11.79)	2.54 (±1.71)	51.04 (±9.68)	57.6 (±71)	8.76 (±5.56)	
Korine et al. (1999)	*R. aegypt.*	*winter*				2.5 (±0.57)	58.08 (±7.04)	75 (±5.8)	8.97 (±1.62)	
		*spring*				2.32 (±0.75)	66 (±10.56)	83.8 (±2)	8.98 (±2.74)	
		*summer*				2.07 (±0.21)	57.2 (±22.88)	79.1 (±5.1)	9.81 (±3.1)	
		*fall*					0.82 (±0.32)	58.96 (±6.16)	78.3 (±3.3)	9.83 (±1.51)	
McLaughlin et al. (2007)	*P. giganteus*					91.8 (±36.3)	2.5 (±0.89)		76 (±70)		
McMichael et al. (2019)	*P. conspicillatus*			562.05		20.99		24.64	78	7.33	
Kishbaugh et al. (2021)	*P. giganteus*						2.79 (±0.86)	83.6 (±27.28)		12.43 (±3.87)^a^	13.22 (±6.16)
Hall et al., (2014)	*P. melanotus natalensis*			1478.56		9.94	0.96	45.76	65.2	3.36	
McMichael et al. (2015)	*P. alecto*			407.46		15.84	1.36		65.5	6.8	
Heard and Whittier, (1997)	*P. vampyrus*			615 (±311)		13 (±13)	4.64 (±8.93)	88 (±52.8)	73 (±3)	8.44 (±1.22)	
	*P. hypomelanus*	*adult*	1023 (±810)		10 (±6)	1.79 (±1.43)	52.8 (±8.8)	74 (±7)	8.55 (±1.2)	
		*juvenile*	2238 (±820)		10 (±9)	1.43 (±0.71)	52.8 (±8.8)	69 (±4)	8.21 (±1.78)	
	*R. rodricensis*			2077 (±2538)		11 (±4)	4.29 (±1.79)	70.4 (±17.6)	69 (±4)	6.49 (±1.83)	
Edson et al. (2018)	*P. poliocephalus*			1100		12.81	1.23	46.64	74.9	7.04	

ALP, alkaline phosphatase; ALT, alanine-aminotransferase; BUN, blood urea nitrogen; CREA, creatinine; TP, total protein; GLU, glucose.

All data are depicted as mean (standard deviation), if available. For comparison, units were adapted if needed.

a Two different devices were used.

b Averages of different populations were calculated.

ALP values could only be determined for the ten available serum samples from *R. aegyptiacus*, since EDTA present in the plasma vials interferes with ALP’s metallic cofactors, resulting in falsely low measured values.

The determined ALT levels showed more variation in *R. aegyptiacus*, but were also significantly higher as compared to values determined for *E. helvum*. The detected values were comparable to previous reports for *R. aegyptiacus* and *E. helvum*, but were lower when compared to other pteropodid bat species. BUN levels were significantly higher in *R. aegyptiacus*, while values determined for *E. helvum* were lower than previously reported for the same species, but comparable to those of other pteropodid species. CREA levels were significantly higher in *R. aegyptiacus* than in *E. helvum*. In this species, generally lower CREA values were found in subadult animals as compared to adult individuals, although these differences turned out to be not significant. Values determined for both species are in accordance with previously reported ranges. We observed significantly higher GLU levels in *E. helvum*, with a wider range of variation as compared to *R. aegyptiacus*. Although not statistically significant, lower GLU values were observed in female and in subadult *E. helvum* and in male *R. aegyptiacus* individuals. Detected values for *R. aegyptiacus* were similar to those reported for other captive bats of the same species, but were lower in comparison to wild *R. aegyptiacus* bats, while values determined for *E. helvum* were lower than those reported for most other species. The TP level was significantly higher in *E. helvum* than in *R. aegyptiacus*. Without reaching statistical significance, values for female *E. helvum* were lower than those in male *E. helvum* bats, and values of subadult *R. aegyptiacus* were lower as compared to those of adults of the same species. In general, the values we determined in our study were lower than in other reports of *R. aegyptiacus* and *E. helvum* and most other pteropodid bat species.

### Hematology

**FIGURE 5:**
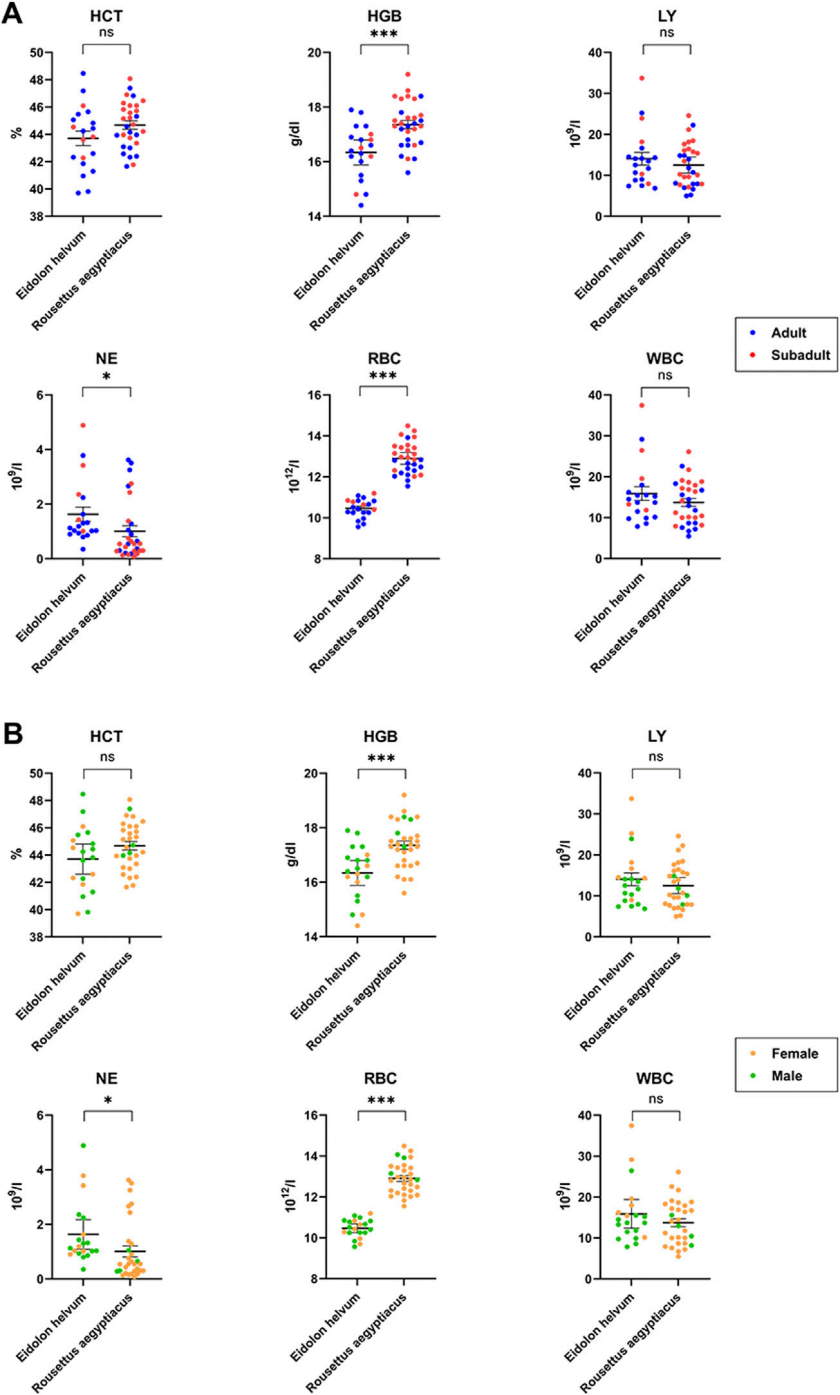
Age- and sex-dependent variations of hematology in *R. aegyptiacus* and *E. helvum*. Single dots represent one animal (adult/subadult) **(A)** and female/male **(B)**. The same cohort was used for comparison. Dotted lines represent the lower detection limit. Solid horizontal bars represent mean ± SEM. Statistically significant differences were determined for hematology parameters (HGB, NE, RBC, HCT, LY, WBC), * = significant: *p*-value < 0.05; *** = extremely or highly significant: *p*-value < 0.001, ns = not statistically significant.

The six parameters HCT, HGB, LY, NE, RBC and WBC were evaluated to establish reference values for *R. aegyptiacus* and *E. helvum* (Supplementary Table S2). These values enable detection of differences between the two species (Table 3) and to investigate potential correlations between the determined levels and age or sex of the animals (Table 3, Figure 5). Reported values were also compared to those previously reported for *R. aegyptiacus* and *E. helvum*, as well as other pteropodid species (Table 4). Results of the other parameters (MON, EO, BA, MCV, MCH, MCHC, PLT, PCT, RDW and PDW) were not considered for statistical analysis, but are supplied in the Supplementary Material (Supplementary Table S3).

**TABLE 3:** Correlation of hematological parameters to age and sex of *R. aegyptiacus* and *E. helvum*.

		*R. aegyptiacus*			*E. helvum*	
		HCT	HGB	RBC	LY	WBC
Sex	Correlation Coefficient	-	-	-	−0.54	−0.57
	Significance (2-tailed)	NS	NS	NS	0.012	0.008
Age	Correlation Coefficient	−0.47	−0.40	−0.42	-	-
	Significance (2-tailed)	0.008	0.028	0.019	NS	NS

Only parameters with a significant correlation to age or sex are depicted (NS: no significant correlation, *p-*value *< 0.05*).

**TABLE 4:** Comparative hematology values determined in pteropodid bat species.

				Parameter							
				HCT (*%*)		HGB (*g/dl*)		LY (*10* ^*9*^ */l*)	NE (*10* ^*9*^ */l*)	WBC (*10* ^*9*^ */l*)	RBC (*10* ^*12*^ */l*)
This paper	*E. helvum*			43.83 (±0.48)		16.38 (±0.2)		14.2 (±1.55)	1.75 (±0.53)	16.55 (±1.83)	10.3 (±0.53)
	*R. aegypt.*			44.67 (±0.31)		17.36 (±0.15)		12.51 (±0.96)	1 (±0.2)	13.71 (±0.97)	12.9 (±0.4)
Selig et al. (2016)	*E. helvum*			42.68 (±19.3)		14.89 (±1.03)		1.7 (±0.8)	1.36 (±1.33)	3.19 (±1.48)	9.47 (±0.7)
Olopade et al. (2020)	*E. helvum1*			39.13		18.13		2.25	1.72	4.64	7.535
Moretti et al. (2021)	*R. aegypt.*			35.5 (±3.1)		12.4 (±1)		1.61 (±0.89)	2.42 (±0.91)	4.2 (±1.4)	9.41 (±0.68)
van der Westhuyzen, (1988)	*R. aegypt.*	*wild m*		43.9 (±2.4)		17.1 (±1.1)				8.8 (±2.5)	11.95 (±0.94)
		*wild f*		57 (±6.2)		17.4 (±2)				8.8 (±5.8)	13.88 (±1.52)
		*captive m*		58.4 (±4.4)		15.6 (±0.8)				12.1 (±4.2)	15.42 (±1.5)
		*captive f*		56.4 (±5.1)		14.4 (±1.4)				10.8 (±3.4)	14.32 (±1.25)
Korine et al. (1999)	*R. aegypt.*	*winter*		55.38 (±3.04)		16.81 (±0.79)					
		*spring*		57.8 (±3.35)		17.42 (±0.69)					
		*summer*		56.6 (±1.94)		17.23 (±0.43)					
		*fall*		52.36 (±2.06)		16.68 (±0.35)					
Noll, (1979)	*R. aegypt.*			50.1		17.4					
Arad and Korine, (1993)	*R. aegypt.*			50.2		17.6					
McMichael et al. (2019)	*P. conspicillatus*			49		16.71		2.5	4.5	7.89	10.13
Kishbaugh et al. (2021)	*P. giganteus* ^b^			47.6 (±3.06)	48.3 (±2.95)	16.19 (±1.04)	16.43 (±0.99)				
Strumpf et al. (2020)	*A. jamaicensis*			53.43 (±2.54)		18.73 (±0.91)		2.56 (±1.99)	1.63 (±0.73)	4.68 (±2.36)	13.56 (±0.73)
Edson et al. (2018)	*P. poliocephalus* ^a^			36.6		15.35		3.26	4.41	7.99	9.53
Hall et al., (2014)	*P. melanotus natalensis*			40.7				4.58	3.74	8.39	
McMichael et al. (2015)	*P. alecto*			47		16.35		1.72	3.57	5.96	9.13
Wightman et al., (1987)	*P. poliocephalus*			47 (±0.7)		17.9 (±1.3)					
Heard and Whittier, (1997)	*P. vampyrus*			44 (±2)		14.6 (±0.9)		8.02 (±2.61)	4.369 (±1.78)	12.55 (±3.24)	8.88 (±0.59)
	*P. hypomelanus*	*adult*	46 (±9)		15.4 (±2.8)		7.56 (±3.93)	3.58 (±1.62)	11.51 (±4.36)	8.7 (±1.32)
		*juvenile*	49 (±8)		15.4 (±1.9)		12.84 (±6.53)	3.11 (±2.04)	16.3 (±5.89)	9.56 (±1.27)
	*P. rodricensis*			43 (±5)		14.2 (±1.6)		1.18 (±0.4)	5.18 (±1.58)	6.46 (±1.71)	7.95 (±0.75)

HCT, hematocrit; HGB, hemoglobin; LY, lymphocytes; NE, neutrophils; WBC, white blood cells; RBC, red blood cells.

All data are depicted as mean ± SD, if available. For comparison, units were adapted if needed.

a Averages of different populations were calculated

b Two different devices were used.

HCT values did not reach statistical significance between the two species or between the sexes. However, an age-dependency was observed in *R. aegyptiacus* where increased levels were observed in subadult animals (OR = 1.03, *p*-value < 0.05). The HCT variation in *E. helvum* was slightly higher as compared to *R. aegyptiacus*. Values of both species are generally comparable to those found in the literature, with those of *R. aegyptiacus* being slightly lower than previously published. HGB levels determined for *R. aegyptiacus* were significantly higher than those observed in *E. helvum,* and were higher in juvenile animals (OR = 1.04, *p*-value < 0.05). Detected values were in accordance to those known from literature. For *E. helvum*, the determined LY values were almost two times higher in females than in males (OR = 1.68, *p*-value < 0.05). No differences were observed between the two species or between the age groups. Lymphocytes were determined to be the dominant white blood cell population (in average about 90%) in both *R. aegyptiacus* and *E. helvum* bats. The levels determined in this study were higher than most values reported for these species in the literature, but were similar to those described for juvenile *Pteropus hypomelanus*. NE levels were significantly higher in *E. helvum* than in *R. aegyptiacus*, while no age- or sex-dependent correlations were detected. In relation to values of other Pteropodidae, values of *R. aegyptiacus* were low. The RBC levels were significantly higher in *R. aegyptiacus,* and were higher in juvenile animals (OR = 1.05, *p*-value < 0.05). In contrast, the RBC variation in *E. helvum* was low. Detected values correlate to previously reported ranges reported for other bat species. The WBC were significantly sex-correlated in *E. helvum* (OR = 1.72, *p*-value < 0.05), with higher values in females. Compared to other pteropodid bat species, WBCs determined in *R. aegyptiacus* and *E. helvum* were relatively high, but were comparable to those reported in a study on captive *R. aegyptiacus* or three different *Pteropus* spp. (van der Westhuyzen, 1988; Heard and Whittier, 1997).

## Discussion

During recent years, the scientific interest in bats has increased considerably, as bats have frequently been identified as reservoirs of emerging infectious disease agents with zoonotic potential. Therefore, unravelling the fundamentals of this reservoir function for highly pathogenic viruses is of particular interest. However, comprehensive datasets on physiological baseline values of the analyzed bat species are largely missing due to a paucity of species-specific tools, limited access to specimens, and high diversity of bat species. Since the majority of species of the suborder *Yinpterochiroptera* are protected by law, and the vast majority of zoonotic viruses have been detected in fruit bats so far, recent studies have utilized fruit bats to monitor responses to viral infections. To enable an accurate interpretation of data and define infection-associated abnormalities, knowledge of physiological baseline values, such as body core temperature and hematology are essential. Overall, our study provides a comprehensive insight into hematological values of healthy *E. helvum* and *R. aegyptiacus* bats, as well as body temperature patterns in *R. aegyptiacus* bats. Both species are kept in breeding colonies at the FLI, which enabled us to determine both inter-species differences, as well as intraspecies sex- and age-related variations.

The temperature profiles of *R. aegyptiacus* determined in our study are in accordance with observations of other captive colonies of the same species (Kulzer, 1963). Similar to our observations, activity and body core temperature increased upon the onset of dusk (≙ lights off in the aviary) and decreased with sunset (≙ illumination of the aviary). The fact that single peaks of high temperature could be traced back to husbandry highlights the necessity to minimize disturbance during phases of sensitive measurements or infection experiments. However, clinical scoring of the animals is inevitable during such experiments and requires veterinary attendance. To circumvent the influence of clinical scoring on measurements of body core temperatures, novel motion monitoring procedures such as video monitoring or digital detection of locomotor activity, which allow clinical scoring without agitating the animals, should be considered (Schutz et al., 2021). We were also able to compare the body temperature parameters in animals kept in the aviary (breeding colony) and in cages (husbandry during infection studies). Although the body core temperature pattern changed slightly once the *R. aegyptiacus* bats were transferred to a cage, the overall association between temperature and circadian rhythm was maintained. This suggests that the dramatic temperature variation of up to 8°C between flying and roosting phases is controlled by circadian processes rather than originating from muscle activity alone. Furthermore, it is suggested that the temperature regulation properties are age-dependent and underdeveloped in subadult individuals. Although this observation could be verified in female *R. aegyptiacus* bats and their pups (Kulzer, 1963; Stones and Wiebers, 1965), we could not detect any significant differences in temperature regulation and age. This can be explained by the fact that the temperature regulation develops very early after birth and has already aligned with adult animals 50 days after birth (Kulzer, 1963), and pups that young were not included in our study. However, as this study was focused on the analysis of tropical fruit bats which do not hibernate, it will prospectively also be of high interest to investigate whether extreme body temperature levels influence the hematological parameters in hibernating bats.

It has been reported that levels of ALP may vary, depending of age, sex, blood type, and possible reproduction status of the individual. Increased levels of ALP in subadult individuals are associated with hepatic and skeletal development and has been reported for bats, e.g. *P. poliocephalus* or *P. hypomelanus* (Jürgens et al., 1981; Arévalo et al., 1987; Wightman et al., 1987; Viljoen et al., 1997; Washington and Van Hoosier, 2012), where adult specimens had only 50% of the ALP level detected in subadults (Heard and Huft, 1998; Edson et al., 2018). However, since no difference between the age groups could be detected in our study, it can be assumed that major maturation processes are already completed in subadult *R. aegyptiacus* bats. The mean value of 381.5 U/L determined for *R. aegyptiacus* in this study is still lower than the levels of up to ∼576 U/L reported in free-ranging, and ∼783 U/L in captive individuals (Cavaleros et al., 2003; Moretti et al., 2021). In contrast, free-ranging *E. helvum* bats had distinctly lower ALP levels than *R. aegyptiacus* (Selig et al., 2016), underlining the necessity to determine the baseline values for each bat species individually. The considerable level of variation of ALP values may be associated with the actual metabolic status of the analyzed animals, since ALP is involved in numerous different metabolic processes (Sharma et al., 2014). It may well be that the lower levels determined in this study are associated with the maintenance and the physiological status of the animals. In contrast, the other reports were either based on free-ranging animals (Cavaleros et al., 2003), or on animals that had been brought into a veterinary clinic a few days before the blood analysis (Moretti et al., 2021). Levels of ALP determined in other free-ranging pteropodid species were generally higher than those determined for *R. aegyptiacus* bats in our study: ALP values in wild bats range from ∼400 U/L (*P. alecto* (McMichael et al., 2015)) to ∼2238 U/L in subadult *P. hypomelanus* and ∼2077 U/L in *P. rodricensis* (Edson et al., 2018). Nevertheless, it can be speculated that ALP values differ greatly amongst species, even within an order. Avian baseline values vary from 24–780 U/L depending on the species, which could be transferable to bats, another order with outstanding diversity.

Values of ALT, CREA, GLU, TP, and BUN in *E. helvum* and *R. aegyptiacus* bats were comparable to reported reference values of other pteropodid bat species. Intriguingly, we observed a noteworthy difference between BUN levels in individuals of the investigated *R. aegyptiacus* colony and free-ranging animals of the same species. This variation might be attributed to the fact that captive individuals do not experience shortage or quality decrease of food and water, compared to wild counterparts (Korine et al., 1999). Therefore, determination of BUN levels during infection experiments serves as valuable readout for behavioral changes, e.g. appetite loss (Korine et al., 1999). However, BUN and CREA levels in *E. helvum*, *R. aegyptiacus* and other bat species are generally lower compared to omnivore or carnivore mammals, which is attributed to the low-protein diet in fruit bats (Heard and Huft, 1998; McLaughlin et al., 2007). Of note, we did not detect considerable differences in GLU levels between male and female *E. helvum* and *R. aegyptiacus* bats. These findings are in line with previous reports regarding sex-related differences in GLU levels of *R. aegyptiacus* (Moretti et al., 2021). However, significantly higher levels of GLU in female compared to male individuals was verified for *Cynopterus sphinx* (Nagarajan-Radha and Devaraj, 2021), a pteropodid bat of similar size and diet like *R. aegyptiacus*. This variation between seemingly closely related species possibly originates from differing sampling timepoints. *C. sphinx* bats were sampled during the roosting period after foraging (Nagarajan-Radha and Devaraj, 2021). *E. helvum* and *R. aegyptiacus* bats in this study were sampled in the morning, prior to being fed. Other reports on GLU levels in captive *R. aegyptiacus*, *P. vampyrus*, and *P. hypomelanus* bats are in line with our findings (Widmaier and Kunz, 1993; Moretti et al., 2021), indicating early sampling timepoints and overall low levels of glucose in the blood. Furthermore, it has been shown that dietary sugars are essential to fuel the high metabolic demands of active flight, since the high levels of GLU rapidly decline during flying phases, as demonstrated in *E. spelaea* and *C. sphinx* (Peng et al., 2017). This feature is specific to fruit bats, since comparative studies of GLU reduction kinetics between fruit bats and insectivorous bats revealed a 4-fold slower decline in the latter (120 min compared to 45 min (Peng et al., 2019)).

The observed variations in the determined clinical chemistry values between the bat species might be explained by differences in immune stati of the animals (especially between wild and captive animals) and the applied methodology, impairing a direct comparison of the data. It has been verified that the method of immobilization (physical restraint, anesthesia) and the type of the analyzer (manual, automatic) may influence the analyte outcome (Widmaier and Kunz, 1993; Heard and Huft, 1998; McMichael et al., 2016; Strumpf et al., 2020). Especially the use of different agents for anesthesia can alter results, as previously reported for different bats (Heard and Huft, 1998; Strumpf et al., 2020). However, since isoflurane-based anesthesia is mild compared to other agents, and even restraining methods can influence the values, this volatile gas was chosen for this study. Isoflurane anesthesia impacts hematology and clinical chemistry values when compared to physical restraint, as demonstrated in other mammals (Welch, 1984; Markovic et al., 1993), birds (Granone et al., 2012; Berg et al., 2019), and even *A. jamaicensis* and *P. hypomelanus* bats (Heard and Huft, 1998; Strumpf et al., 2020). However, e.g., ketamine-xylazine-based anesthesia is known to have even higher impacts on the results (Widmaier and Kunz, 1993) and since isoflurane mainly acts on neuronal function, the restraining effects on leukocytes are assumed to be diminished quickly. Ultimately, this verifies the necessity to establish baseline values for clinical chemistry and hematology of isolated colonies, as well as the analyzers used in the respective study.

Hematological analyses of *E. helvum* and *R. aegyptiacus* bats of different sexes and ages revealed striking differences. Firstly, we report that *E. helvum* bats had twice as many circulating neutrophils compared to *R. aegyptiacus* bats. This observation is in contrast to previous reports of NE levels in these species, since a comparison of these independent studies revealed inverse NE levels in *E. helvum* and *R. aegyptiacus* (Selig et al., 2016; Olopade et al., 2020; Moretti et al., 2021). However, it has to be considered that especially levels of circulating NEs may differ during seasons, hibernation (Wołk and Bogdanowicz, 1987), and migration (e.g., *Pipistrellus nathusii (* *Voigt et al., 2020* *)*), specifically in species inhabiting temperate areas. Strikingly, we report 10-fold higher numbers of circulating lymphocytes in both species, compared to other hematological reports in the same or other pteropodid bat species. It has been demonstrated that lymphocytes are the dominating leukocyte subset in the blood of pteropodid bats, e.g., *P. vampyrus* and *P. hypomelanus* (Heard and Huft, 1998). We observed the same ratio in *E. helvum* and *R. aegyptiacus* bats, although in higher abundance. Interestingly, lymphocytes, and therefore WBCs in general, were significantly enriched in female *E. helvum* bats, but no sex-related difference could be detected in *R. aegyptiacus*. This suggests species-specific differences rather than different immune stati within the colony (due to e.g., pregnancy (Kühnert et al., 1998)), since leukocyte enrichment in females has been described in free-ranging *E. helvum, P. natalensis,* and *P. poliocephalus* bats (Hall et al., 2014; Edson et al., 2018; Olopade et al., 2020). Although variations linked to immune-senescence and -maturation in circulating leukocytes have been validated in several chiropteran species, e.g., *P. giganteus* (McLaughlin et al., 2007; Schneeberger et al., 2014) and *P. poliocephalus* (Edson et al., 2018) and other mammals (Aminzadeh and Parsa, 2011), we could not detect age-related differences for *E. helvum* or *R. aegyptiacus*. Interestingly, it has been recently demonstrated that the number of circulating immune cells (especially neutrophils and lymphocytes) is size-independent in bats and did not differ significantly among 64 different bat species (Cornelius Ruhs et al., 2021). Bats are the only mammals capable of active flight, which represents a demanding way of locomotion with a high oxygen requirement. This results in elevated levels of HGB, HCT, and the number of RBCs in bats in comparison to non-flying mammals of similar size (Jürgens et al., 1981; Arévalo et al., 1987; Wightman et al., 1987; Viljoen et al., 1997). The values determined for these three parameters were comparable between captive *E. helvum* and *R. aegyptiacus*, and were in the same range as determined for other captive colonies of the same species or even free-ranging pteropodid bats (Noll, 1979; van der Westhuyzen, 1988; Arad and Korine, 1993; Heard and Whittier, 1997; Korine et al., 1999; Selig et al., 2016; Olopade et al., 2020; Moretti et al., 2021). Furthermore, similar values were observed in insectivorous bats (Woz.xl et al., 1987; Bandouchova et al., 2020; Paksuz, 2021), indicating conserved features. A study restricting water intake upon heat exposure in *R. aegyptiacus* bats reported no change in HGB, HCT and total protein (TP), which further demonstrates a crucial role for stability of these values (Arad and Korine, 1993). Interestingly, while sex-related differences have been reported for *P. alecto* and *P. conspicillatus* (McMichael et al., 2019), we report age-related differences for captive *E. helvum* and *R. aegyptiacus* bats. While HCT values were enriched in juvenile *R. aegyptiacus*, HGB values were enriched in juvenile *E. helvum* bats, compared to adult counterparts. These differences possibly result from varying population dynamics among captive and free-ranging individuals, as well as nutrient availability and parasite burden (McMichael et al., 2019).

Similar to the capabilities of temperature regulation in adult individuals and pups, clinical chemistry and hematology are also influenced by maturation processes, e.g. in *Antrozous pallidus* (Bassett and Wiederhielm, 1984) and *R. aegyptiacus* (Moretti et al., 2021). Although newborn bats already reach a blood composition in a similar range as adults a few weeks after birth (40–70 days), maturation processes like skeletal growth and immune maturation should be considered during baseline establishment or infection studies. Of note, is has been demonstrated previously that juvenile *R. aegyptiacus* bats play a crucial role for virus maintenance and spread in a colony, e.g., MARV (Amman et al., 2012). This further highlights the necessity of baseline establishment for parameters in bats of various ages and sexes to accurately predict zoonotic potential and spillover risks, as for example the elevated WBC level in juvenile bats may contribute to their role in virus transmission. Since bats only rarely develop apparent clinical signs, changes in hematology and clinical chemistry might be mostly minor, yet crucial for disease tolerance, as demonstrated in *P. alecto* (McMichael et al., 2017). Therefore, precise and comprehensive baseline values for different bat species are essential to define pathogen-associated changes.

With this study, we provide a comprehensive analysis for captive *E. helvum* and *R. aegyptiacus* bats. Taking previous studies into account, it is evident that blood parameters vary between captive and free-ranging, as well as between different captive colonies of *E. helvum* and *R. aegyptiacus*. By delineating age and sex, we established baseline values for four different groups in each species, which is essential considering different roles of each group within the colony. Since hematology and clinical chemistry are affected by sex and maturation processes, definition of separate baseline values significantly increases the accuracy of clinical scoring.

## Data Availability

The original contributions presented in the study are included in the article/Supplementary Material, further inquiries can be directed to the corresponding author.

## References

[B1] Ahn M. Cui J. Irving A. T. Wang L.-F. (2016). Unique Loss of the PYHIN Gene Family in Bats Amongst Mammals: Implications for Inflammasome Sensing. Sci. Rep. 6, 21722. 10.1038/srep21722 26906452 PMC4764838

[B2] Ahn M. Anderson D. E. Zhang Q. Tan C. W. Lim B. L. Luko K. (2019). Dampened NLRP3-Mediated Inflammation in Bats and Implications for a Special Viral Reservoir Host. Nat. Microbiol. 4 (5), 789–799. 10.1038/s41564-019-0371-3 30804542 PMC7096966

[B3] Aminzadeh Z. Parsa E. (2011). Relationship between Age and Peripheral White Blood Cell Count in Patients with Sepsis. Int. J. Prev. Med. 2 (4), 238–242. 22174963 PMC3237266

[B4] Amman B. R. Carroll S. A. Reed Z. D. Sealy T. K. Balinandi S. Swanepoel R. (2012). Seasonal Pulses of Marburg Virus Circulation in Juvenile *Rousettus Aegyptiacus* Bats Coincide with Periods of Increased Risk of Human Infection. PLoS Pathog. 8 (10), e1002877. 10.1371/journal.ppat.1002877 23055920 PMC3464226

[B5] Amman B. R. Albariño C. G. Bird B. H. Nyakarahuka L. Sealy T. K. Balinandi S. (2015). A Recently Discovered Pathogenic Paramyxovirus, Sosuga Virus, Is Present in *Rousettus Aegyptiacus* Fruit Bats at Multiple Locations in Uganda. J. Wildl. Dis. 51 (3), 774–779. 10.7589/2015-02-044 25919464 PMC5022529

[B6] Amman B. R. Schuh A. J. Sealy T. K. Spengler J. R. Welch S. R. Kirejczyk S. G. M. (2020). Experimental Infection of Egyptian Rousette Bats (*Rousettus Aegyptiacus*) with Sosuga Virus Demonstrates Potential Transmission Routes for a Bat-Borne Human Pathogenic Paramyxovirus. PLoS Negl. Trop. Dis. 14 (3), e0008092. 10.1371/journal.pntd.0008092 32119657 PMC7067492

[B7] Arad Z. Korine C. (1993). Effect of Water Restriction on Energy and Water Balance and Osmoregulation of the Fruit Bat *Rousettus Aegyptiacus* . J. Comp. Physiol. B 163 (5), 401–405. 10.1007/BF00265645 8254118

[B8] Arévalo F. Pérez-Suárez G. López-Luna P. (1987). Hematological Data and Hemoglobin Components in Bats (Vespertilionidae). Comp. Biochem. Physiol. Part A Physiol. 88 (3), 447–450. 10.1016/0300-9629(87)90061-2 2892619

[B9] Baker K. S. Todd S. Marsh G. Fernandez-Loras A. Suu-Ire R. Wood J. L. N. (2012). Co-circulation of Diverse Paramyxoviruses in an Urban African Fruit Bat Population. J. Gen. Virol. 93 (Pt 4), 850–856. 10.1099/vir.0.039339-0 22205718 PMC3542712

[B10] Balkema-Buschmann A. Rissmann M. Kley N. Ulrich R. Eiden M. Groschup M. H. (2018). Productive Propagation of Rift Valley Fever Phlebovirus Vaccine Strain MP-12 in *Rousettus Aegyptiacus* Fruit Bats. Viruses 10 (12), 681. 10.3390/v10120681 PMC6315703 30513679

[B11] Bandouchova H. Zukal J. Linhart P. Berkova H. Brichta J. Kovacova V. (2020). Low Seasonal Variation in Greater Mouse-Eared Bat (*Myotis myotis*) Blood Parameters. PLoS One 15 (7), e0234784. 10.1371/journal.pone.0234784 32634149 PMC7340307

[B12] Bassett J. E. Wiederhielm C. A. (1984). Postnatal Changes in Hematology of the Bat Antrozous Pallidus. Comp. Biochem. Physiol. Part A Physiol. 78 (4), 737–742. 10.1016/0300-9629(84)90625-x 6149046

[B13] Begeman L. Suu-Ire R. Banyard A. C. Drosten C. Eggerbauer E. Freuling C. M. (2020). Experimental Lagos Bat Virus Infection in Straw-Colored Fruit Bats: A Suitable Model for Bat Rabies in a Natural Reservoir Species. PLoS Negl. Trop. Dis. 14 (12), e0008898. 10.1371/journal.pntd.0008898 33320860 PMC7771871

[B14] Berg K. J. Whittington J. K. Watson M. K. Wiggen K. Blalock A. Mitchell M. A. (2019). Effects of Isoflurane Anesthesia on the Hematologic Values of Rehabilitated Wild Owls. J. Avian Med. Surg. 33 (4), 369–380. 10.1647/2017-333 31833305

[B15] Bondet V. Le Baut M. Le Poder S. Lécu A. Petit T. Wedlarski R. (2021). Constitutive IFNα Protein Production in Bats. Front. Immunol. 12, 735866. 10.3389/fimmu.2021.735866 34790193 PMC8591296

[B16] Calisher C. H. Childs J. E. Field H. E. Holmes K. V. Schountz T. (2006). Bats: Important Reservoir Hosts of Emerging Viruses. Clin. Microbiol. Rev. 19 (3), 531–545. 10.1128/CMR.00017-06 16847084 PMC1539106

[B17] Cavaleros M. Buffenstein R. Patrick Ross F. Pettifor J. M. (2003). Vitamin D Metabolism in a Frugivorous Nocturnal Mammal, the Egyptian Fruit Bat (*Rousettus Aegyptiacus*). General Comp. Endocrinol. 133 (1), 109–117. 10.1016/s0016-6480(03)00150-3 12899852

[B18] Chionh Y. T. Cui J. Koh J. Mendenhall I. H. Ng J. H. J. Low D. (2019). High Basal Heat-Shock Protein Expression in Bats Confers Resistance to Cellular Heat/oxidative Stress. Cell Stress Chaper. 24 (4), 835–849. 10.1007/s12192-019-01013-y PMC6629734 31230214

[B19] Cornelius Ruhs E. Becker D. J. Oakey S. J. Ogunsina O. Fenton M. B. Simmons N. B. (2021). Body Size Affects Immune Cell Proportions in Birds and Non-volant Mammals, but Not Bats. J. Exp. Biol. 224 (13), 1–16. 10.1242/jeb.241109 34104965

[B20] Cotterill F. (2001). New Records for Two Species of Fruit Bats (Megachiroptera: Mammalia) in Southeast Africa, with Taxonomic Comments. Durb. Mus. Novit. 26, 53–56.

[B21] David D. Davidson I. Berkowitz A. Karniely S. Edery N. Bumbarov V. (2020). A Novel Poxvirus Isolated from an Egyptian Fruit Bat in Israel. Vet. Med. Sci. 6 (3), 587–590. 10.1002/vms3.233 32100464 PMC7397903

[B22] Edson D. Field H. McMichael L. Mayer D. Martin J. Welbergen J. (2018). Hematology, Plasma Biochemistry, and Urinalysis of Free-Ranging Grey-Headed Flying Foxes ( Pteropus Poliocephalus) in Australia. J. Zoo Wildl. Med. 49 (3), 591–598. 10.1638/2017-0126.1 30212311

[B23] Fagre A. C. Lewis J. Miller M. R. Mossel E. C. Lutwama J. J. Nyakarahuka L. (2021). Subgenomic Flavivirus RNA (sfRNA) Associated with Asian Lineage Zika Virus Identified in Three Species of Ugandan Bats (*Family Pteropodidae*). Sci. Rep. 11 (1), 8370. 10.1038/s41598-021-87816-5 33863991 PMC8052318

[B24] Fahr J. Abedi-Lartey M. Esch T. Machwitz M. Suu-Ire R. Wikelski M. (2015). Pronounced Seasonal Changes in the Movement Ecology of a Highly Gregarious Central-Place Forager, the African Straw-Coloured Fruit Bat (*Eidolon Helvum*). PLoS One 10 (10), e0138985. 10.1371/journal.pone.0138985 26465139 PMC4605647

[B25] Field H. Young P. Yob J. M. Mills J. Hall L. Mackenzie J. (2001). The Natural History of Hendra and Nipah Viruses. Microbes Infect. 3 (4), 307–314. 10.1016/s1286-4579(01)01384-3 11334748

[B26] Fumagalli M. R. Zapperi S. La Porta C. A. M. (2021). Role of Body Temperature Variations in Bat Immune Response to Viral Infections. J. R. Soc. Interface. 18 (180), 20210211. 10.1098/rsif.2021.0211 34314652 PMC8315833

[B27] George D. B. Webb C. T. Farnsworth M. L. O'Shea T. J. Bowen R. A. Smith D. L. (2011). Host and Viral Ecology Determine Bat Rabies Seasonality and Maintenance. Proc. Natl. Acad. Sci. U.S.A. 108 (25), 10208–10213. 10.1073/pnas.1010875108 21646516 PMC3121824

[B28] Granone T. D. de Francisco O. N. Killos M. B. Quandt J. E. Mandsager R. E. Graham L. F. (2012). Comparison of Three Different Inhalant Anesthetic Agents (Isoflurane, Sevoflurane, Desflurane) in Red-Tailed Hawks (*Buteo jamaicensis*). Veterinary Anaesth. Analgesia 39 (1), 29–37. 10.1111/j.1467-2995.2011.00668.x 22103452

[B29] Hall J. Rose K. Smith C. De Jong C. Phalen D. Austen J. (2014). Health Assessment of the Christmas Island Flying Fox (Pteropus Melanotus Natalis). J. Wildl. Dis. 50 (3), 447–458. 10.7589/2013-09-245 24807172

[B30] Halpin K. Young P. L. Field H. E. Mackenzie J. S. (2000). Isolation of Hendra Virus from Pteropid Bats: a Natural Reservoir of Hendra Virus. J. Gen. Virol. 81 (Pt 8), 1927–1932. 10.1099/0022-1317-81-8-1927 10900029

[B31] Halwe N. J. Gorka M. Hoffmann B. Rissmann M. Breithaupt A. Schwemmle M. (2021). Egyptian Fruit Bats (*Rousettus Aegyptiacus*) Were Resistant to Experimental Inoculation with Avian-Origin Influenza A Virus of Subtype H9N2, but Are Susceptible to Experimental Infection with Bat-Borne H9N2 Virus. Viruses 13 (4), 672. 10.3390/v13040672 33919890 PMC8070959

[B32] Han H.-J. Wen H.-l. Zhou C.-M. Chen F.-F. Luo L.-M. Liu J.-w. (2015). Bats as Reservoirs of Severe Emerging Infectious Diseases. Virus Res. 205, 1–6. 10.1016/j.virusres.2015.05.006 25997928 PMC7132474

[B33] Hayman D. T. S. Suu-Ire R. Breed A. C. McEachern J. A. Wang L. Wood J. L. N. (2008). Evidence of Henipavirus Infection in West African Fruit Bats. PLoS One 3 (7), e2739. 10.1371/journal.pone.0002739 18648649 PMC2453319

[B34] Hayman D. T. S. Emmerich P. Yu M. Wang L.-F. Suu-Ire R. Fooks A. R. (2010). Long-term Survival of an Urban Fruit Bat Seropositive for Ebola and Lagos Bat Viruses. PLoS One 5 (8), e11978. 10.1371/journal.pone.0011978 20694141 PMC2915915

[B35] Hayman D. T. S. Yu M. Crameri G. Wang L.-F. Suu-Ire R. Wood J. L. N. (2012). Ebola Virus Antibodies in Fruit Bats, Ghana, West Africa. Emerg. Infect. Dis. 18 (7), 1207–1209. 10.3201/eid1807.111654 22710257 PMC3376795

[B36] Hayman D. T. S. (2016). As the Bat Flies. Science 354 (6316), 1099–1100. 10.1126/science.aaj1818 27934719

[B37] Heard D. J. Huft V. J. (1998). The Effects of Short-Term Physical Restraint and Isoflurane Anesthesia on Hematology and Plasma Biochemistry in the Island Flying Fox (*Pteropus Hypomelanus*). J. Zoo. Wildl. Med. 29 (1), 14–17. 9638618

[B38] Heard D. J. Whittier D. A. (1997). Hematologic and Plasma Biochemical Reference Values for Three Flying Fox Species (Pteropus sp.). J. Zoo. Wildl. Med. 28 (4), 464–470. 9523641

[B39] Irving A. T. Ahn M. Goh G. Anderson D. E. Wang L.-F. (2021). Lessons from the Host Defences of Bats, a Unique Viral Reservoir. Nature 589 (7842), 363–370. 10.1038/s41586-020-03128-0 33473223

[B40] Jacobsen N. H. G. Du Plessis E. (1976). Observations on the Ecology and Biology of the Cape Fruit Bat Rousettus Aegyptiacus Leachi in the Eastern Transvaal. South Afr. J. Sci. 72, 270–273.

[B41] Jacobsen N. H. G. J. Ferguson W. (1985). Radio Tracking of Problem Fruit Bats (Rousettus Aegyptiacus) in the Transvaal with Notes on Flight and Energetics. Z. für Säugetierkd. im Auftrage Dtsch. Ges. für Säugetierkd. e.V. 51, 205–208.

[B42] Johnson N. Vos A. Freuling C. Tordo N. Fooks A. R. Müller T. (2010). Human Rabies Due to Lyssavirus Infection of Bat Origin. Veter. Microbiol. 142 (3-4), 151–159. 10.1016/j.vetmic.2010.02.001 20188498

[B43] Jones M. Schuh A. Amman B. Sealy T. Zaki S. Nichol S. (2015). Experimental Inoculation of Egyptian Rousette Bats (Rousettus Aegyptiacus) with Viruses of the Ebolavirus and Marburgvirus Genera. Viruses 7 (7), 3420–3442. 10.3390/v7072779 26120867 PMC4517108

[B44] Jones M. Amman B. Sealy T. Uebelhoer L. Schuh A. Flietstra T. (2019). Clinical, Histopathologic, and Immunohistochemical Characterization of Experimental Marburg Virus Infection in A Natural Reservoir Host, the Egyptian Rousette Bat (*Rousettus Aegyptiacus*). Viruses 11 (3), 214. 10.3390/v11030214 PMC6466277 30832364

[B45] Jürgens K. D. Bartels H. Bartels R. (1981). Blood Oxygen Transport and Organ Weights of Small Bats and Small Non-flying Mammals. Respir. Physiol. 45 (3), 243–260. 10.1016/0034-5687(81)90009-8 7330485

[B46] Kingdon J. (1984). East African Mammals : An Atlas of Evolution in Africa. Chicago, London: University of Chicago Press Academic.

[B47] Kishbaugh J. C. Valitutto M. T. Aung O. Naing Tun K. Y. Hayek L. C. Yu J. H. (2021). Use of a Portable Analyzer for Venous Blood Gas and Biochemistry Analysis in Free-Ranging Indian Flying Foxes (*Pteropus giganteus*) in Myanmar. J. Wildl. Dis. 57 (1), 242–245. 10.7589/JWD-D-20-00095 33635999

[B48] Korine C. Zinder O. Arad Z. (1999). Diurnal and Seasonal Changes in Blood Composition of the Free-Living Egyptian Fruit Bat (Rousettus Aegyptiacus). J. Comp. Physiol. B Biochem. Syst. Environ. Physiol. 169 (4-5), 280–286. 10.1007/s003600050222 10466219

[B49] Kühnert M. Strohmeier R. Stegmüller M. Halberstadt E. (1998). Changes in Lymphocyte Subsets during Normal Pregnancy. Eur. J. Obstet. Gynecol. Reproduct. Biol. 76 (2), 147–151. 10.1016/s0301-2115(97)00180-2 9481564

[B50] Kulzer E. (1963). Temperaturregulation bei Flughunden der Gattung Rousettus Gray. Z. Vergl. Physiol. 46 (6), 595–618. 10.1007/bf00298161

[B51] Kwiecinski G. G. Griffiths T. A. (1999). Rousettus Egyptiacus. Mamm. Species 611, 1. 10.2307/3504411

[B52] Markotter W. Geldenhuys M. Jansen van Vuren P. Kemp A. Mortlock M. Mudakikwa A. (2019). Paramyxo- and Coronaviruses in Rwandan Bats. TropicalMed 4 (3), 99. 10.3390/tropicalmed4030099 PMC6789848 31269631

[B53] Markovic S. N. Knight P. R. Murasko D. M. (1993). Inhibition of Interferon Stimulation of Natural Killer Cell Activity in Mice Anesthetized with Halothane or Isoflurane. Anesthesiology 78 (4), 700–706. 10.1097/00000542-199304000-00013 8466070

[B54] McLaughlin A. B. Epstein J. H. Prakash V. Smith C. S. Daszak P. Field H. E. (2007). Plasma Biochemistry and Hematologic Values for Wild-Caught Flying Foxes (*Pteropus giganteus*) in India. J. Zoo Wildl. Med. 38 (3), 446–452. 10.1638/1042-7260(2007)38[446:pbahvf]2.0.co;2 17939354

[B55] McMichael L. Edson D. McLaughlin A. Mayer D. Kopp S. Meers J. (2015). Haematology and Plasma Biochemistry of Wild Black Flying-Foxes, (Pteropus Alecto) in Queensland, Australia. PLoS One 10 (5), e0125741. 10.1371/journal.pone.0125741 25938493 PMC4418720

[B56] McMichael L. Edson D. Mayer D. McLaughlin A. Goldspink L. Vidgen M. E. (2016). Temporal Variation in Physiological Biomarkers in Black Flying-Foxes (Pteropus Alecto), Australia. Ecohealth 13 (1), 49–59. 10.1007/s10393-016-1113-0 27026357 PMC7087910

[B57] McMichael L. Edson D. Mayer D. Broos A. Kopp S. Meers J. (2017). Physiologic Biomarkers and Hendra Virus Infection in Australian Black Flying Foxes (Pteropus Alecto). J. Wildl. Dis. 53 (1), 111–120. 10.7589/2016-05-100 27723384

[B58] McMichael L. Edson D. McKeown A. Sánchez C. Mayer D. Kopp S. (2019). Hematology and Plasma Biochemistry of Wild Spectacled Flying Foxes ( Pteropus Conspicillatus) in Australia. J. Wildl. Dis. 55 (2), 449–454. 10.7589/2018-04-096 30325258

[B59] Minematsu S. Watanabe M. Tsuchiya N. Watanabe M. Amagaya S. (1995). Diurnal Variations in Blood Chemical Items in Sprague-Dawley Rats. Exp. Anim. 44 (3), 223–232. 10.1538/expanim.44.223 7556424

[B60] Monadjem A. Taylor P. J. Cotterill F. Schoeman M. C. (2020). Bats of Southern and Central Africa : A Biogeographic and Taxonomic Synthesis. Johannesburg: Wits University Press.

[B61] Moratelli R. Calisher C. H. (2015). Bats and Zoonotic Viruses: Can We Confidently Link Bats with Emerging Deadly Viruses? Mem. Inst. Oswaldo Cruz 110 (1), 1–22. 10.1590/0074-02760150048 PMC4371215 25742261

[B62] Moretti P. Ravasio G. Magnone W. Di Cesare F. Paltrinieri S. Pecile A. (2021). Haematological, Serum Biochemical and Electrophoretic Data on Healthy Captive Egyptian Fruit Bats (*Rousettus Aegyptiacus*). Lab. Anim. 55 (2), 158–169. 10.1177/0023677220948542 32838610

[B63] Muller M. A. Devignot S. Lattwein E. Corman V. M. Maganga G. D. Gloza-Rausch F. (2016). Evidence for Widespread Infection of African Bats with Crimean-Congo Hemorrhagic Fever-like Viruses. Sci. Rep. 6, 26637. 10.1038/srep26637 27217069 PMC4877572

[B64] Nagarajan-Radha V. Devaraj P. S. D. (2021). Sex Differences in Postprandial Blood Glucose and Body Surface Temperature Are Contingent on Flight in the Fruit Bat, *Cynopterus Sphinx* . Biol. Open 10 (2), bio053926. 10.1242/bio.053926 33509836 PMC7903995

[B65] Noll U. G. (1979). Body Temperature, Oxygen Consumption, Noradrenaline Response and Cardiovascular Adaptations in the Flying Fox, Rousettus Aegyptiacus. Comp. Biochem. Physiol. Part A Physiol. 63, 79–88. 10.1016/0300-9629(79)90631-5

[B66] Ogawa H. Kajihara M. Nao N. Shigeno A. Fujikura D. Hang’ombe B. (2017). Characterization of a Novel Bat Adenovirus Isolated from Straw-Colored Fruit Bat (Eidolon Helvum). Viruses 9 (12), 371. 10.3390/v9120371 PMC5744146 29207524

[B67] Okia N. O. (1987). Reproductive Cycles of East African Bats. J. Mammal. 68 (1), 138–141. 10.2307/1381058

[B68] Olopade J. O. Anosike F. Lanipekun D. O. Adebiyi O. E. Ogunsuyi O. M. Bakare A. A. (2020). Haematological Studies and Micronucleus Assay of Straw-Coloured Fruit Bats (Eidolon Helvum). Niger. J. Physiol. Sci. 35 (2), 181–186. 34009208

[B69] O’Shea T. J. Cryan P. M. Cunningham A. A. Fooks A. R. Hayman D. T. S. Luis A. D. (2014). Bat Flight and Zoonotic Viruses. Emerg. Infect. Dis. 20 (5), 741–745. 10.3201/eid2005.130539 24750692 PMC4012789

[B70] Paksuz E. P. (2021). Hematology and Plasma Biochemistry of Wild-Caught Greater Mouse-Eared Bat Myotis Myotis (Chiroptera: Vespertilionidae). Trakya Univ. J. Nat. Sci. 10.23902/trkjnat.962609

[B71] Paweska J. T. Jansen van Vuren P. Masumu J. Leman P. A. Grobbelaar A. A. Birkhead M. (2012). Virological and Serological Findings in Rousettus Aegyptiacus Experimentally Inoculated with Vero Cells-Adapted Hogan Strain of Marburg Virus. PLoS One 7 (9), e45479. 10.1371/journal.pone.0045479 23029039 PMC3444458

[B72] Paweska J. Storm N. Grobbelaar A. Markotter W. Kemp A. Jansen van Vuren P. (2016). Experimental Inoculation of Egyptian Fruit Bats (*Rousettus Aegyptiacus*) with Ebola Virus. Viruses 8 (2), 29. 10.3390/v8020029 PMC4776184 26805873

[B73] Peng X. He X. Liu Q. Sun Y. Liu H. Zhang Q. (2017). Flight Is the Key to Postprandial Blood Glucose Balance in the Fruit Bats Eonycteris Spelaea and *Cynopterus Sphinx* . Ecol. Evol. 7 (21), 8804–8811. 10.1002/ece3.3416 29152179 PMC5677482

[B74] Peng X. He X. Sun Y. Liang J. Xie H. Wang J. (2019). Difference in Glucose Tolerance between Phytophagous and Insectivorous Bats. J. Comp. Physiol. B 189 (6), 751–756. 10.1007/s00360-019-01242-8 31691155

[B75] Plowright R. K. Eby P. Hudson P. J. Smith I. L. Westcott D. Bryden W. L. (2015). Ecological Dynamics of Emerging Bat Virus Spillover. Proc. R. Soc. B 282 (1798), 20142124. 10.1098/rspb.2014.2124 PMC4262174 25392474

[B76] Powers D. R. Tobalske B. W. Wilson J. K. Woods H. A. Corder K. R. (2015). Heat Dissipation during Hovering and Forward Flight in Hummingbirds. R. Soc. open Sci. 2 (12), 150598. 10.1098/rsos.150598 27019742 PMC4807464

[B77] Powers D. R. Langland K. M. Wethington S. M. Powers S. D. Graham C. H. Tobalske B. W. (2017). Hovering in the Heat: Effects of Environmental Temperature on Heat Regulation in Foraging Hummingbirds. R. Soc. open Sci. 4 (12), 171056. 10.1098/rsos.171056 29308244 PMC5750011

[B78] Racey P. (2004). 8,000,000 Fruit Bats: Africa’s Best-Kept Wildlife Secret. Bats 22 (1), 1–5.

[B79] Richter H. V. Cumming G. S. (2008). First Application of Satellite Telemetry to Track African Straw‐coloured Fruit Bat Migration. J. Zool. 275 (2), 172–176. 10.1111/j.1469-7998.2008.00425.x

[B80] Rummel A. D. Swartz S. M. Marsh R. L. (2019). Warm Bodies, Cool Wings: Regional Heterothermy in Flying Bats. Biol. Lett. 15 (9), 20190530. 10.1098/rsbl.2019.0530 31506035 PMC6769150

[B81] Schlottau K. Rissmann M. Graaf A. Schön J. Sehl J. Wylezich C. (2020). SARS-CoV-2 in Fruit Bats, Ferrets, Pigs, and Chickens: an Experimental Transmission Study. Lancet Microbe 1 (5), e218–e225. 10.1016/s2666-5247(20)30089-6 32838346 PMC7340389

[B82] Schneeberger K. Courtiol A. Czirják G. Á. Voigt C. C. (2014). Immune Profile Predicts Survival and Reflects Senescence in a Small, Long-Lived Mammal, the Greater Sac-Winged Bat (*Saccopteryx Bilineata*). PLoS One 9 (9), e108268. 10.1371/journal.pone.0108268 25254988 PMC4177908

[B83] Schuh A. J. Amman B. R. Sealy T. K. Spengler J. R. Nichol S. T. Towner J. S. (2017). Egyptian Rousette Bats Maintain Long-Term Protective Immunity against Marburg Virus Infection Despite Diminished Antibody Levels. Sci. Rep. 7 (1), 8763. 10.1038/s41598-017-07824-2 28821722 PMC5562751

[B84] Schutz A. K. Schöler V. Krause E. T. Fischer M. Müller T. Freuling C. M. (2021). Application of YOLOv4 for Detection and Motion Monitoring of Red Foxes. Animals 11 (6), 1723. 10.3390/ani11061723 34207726 PMC8228056

[B85] Seifert S. N. Letko M. C. Bushmaker T. Laing E. D. Saturday G. Meade-White K. (2020). *Rousettus Aegyptiacus* Bats Do Not Support Productive Nipah Virus Replication. J. Infect. Dis. 221 (Suppl. 4), S407–S413. 10.1093/infdis/jiz429 31682727 PMC7199784

[B86] Selig M. Lewandowski A. Kent M. S. (2016). Establishment of Reference Intervals for Hematology and Biochemistry Analytes in a Captive Colony of Straw-Colored Fruit Bats (Eidolon Helvum). J. Zoo Wildl. Med. 47 (1), 106–112. 10.1638/2015-0040.1 27010270

[B87] Sharma U. Pal D. Prasad R. (2014). Alkaline Phosphatase: an Overview. Ind. J. Clin. Biochem. 29 (3), 269–278. 10.1007/s12291-013-0408-y PMC4062654 24966474

[B88] Sørensen U. G. Halberg K. (2001). Mammoth Roost of Nonbreeding Straw-Coloured Fruit Bat Eidolon Helvum (Kerr, 1792) in Zambia. Afr. J. Ecol. 39 (2), 213–215. 10.1046/j.1365-2028.2000.00281.x

[B89] Solari S. Baker R. J. (2007). Mammal Species of the World: a Taxonomic and Geographic Reference. J. Mammal. 88 (3), 824–830. 10.1644/06-mamm-r-422.1

[B90] Stones R. C. Wiebers J. E. (1965). A Review of Temperature Regulation in Bats (Chiroptera). Am. Midl. Nat. 74 (1), 155. 10.2307/2423129

[B91] Strumpf A. A. Malmlov A. Ayers J. D. Schountz T. Kendall L. V. (2020). Hematologic Values of Jamaican Fruit Bats (*Artibeus jamaicensis*) and the Effects of Isoflurane Anesthesia. J. Am. Assoc. Lab. Anim. Sci. 59 (3), 275–281. 10.30802/AALAS-JAALAS-19-000056 32164795 PMC7210728

[B92] Subudhi S. Rapin N. Misra V. (2019). Immune System Modulation and Viral Persistence in Bats: Understanding Viral Spillover. Viruses 11 (2), 192. 10.3390/v11020192 PMC6410205 30813403

[B93] Suu-Ire R. Fooks A. Banyard A. Selden D. Amponsah-Mensah K. Riesle S. (2017). Lagos Bat Virus Infection Dynamics in Free-Ranging Straw-Colored Fruit Bats (Eidolon Helvum). TropicalMed 2 (3), 25. 10.3390/tropicalmed2030025 PMC6082102 30270883

[B94] Swanepoel R. Smit S. B. Rollin P. E. Formenty P. Leman P. A. Kemp A. (2007). Studies of Reservoir Hosts for Marburg Virus. Emerg. Infect. Dis. 13 (12), 1847–1851. 10.3201/eid1312.071115 18258034 PMC2876776

[B95] Thomas S. P. Suthers R. A. (1972). The Physiology and Energetics of Bat Flight. J. Exp. Biol. 57 (2), 317–335. 10.1242/jeb.57.2.317

[B96] Towner J. S. Amman B. R. Sealy T. K. Carroll S. A. R. Comer J. A. Kemp A. (2009). Isolation of Genetically Diverse Marburg Viruses from Egyptian Fruit Bats. PLoS Pathog. 5 (7), e1000536. 10.1371/journal.ppat.1000536 19649327 PMC2713404

[B97] Tsoar A. Nathan R. Bartan Y. Vyssotski A. Dell'Omo G. Ulanovsky N. (2011). Large-scale Navigational Map in a Mammal. Proc. Natl. Acad. Sci. U.S.A. 108 (37), E718–E724. 10.1073/pnas.1107365108 21844350 PMC3174628

[B98] van der Westhuyzen J. (1988). Haematology and Iron Status of the Egyptian Fruit Bat, *Rousettus Aegyptiacus* . Comp. Biochem. Physiol. Part A Physiol. 90 (1), 117–120. 10.1016/0300-9629(88)91015-8 2900095

[B99] van Doremalen N. Schäfer A. Menachery V. Letko M. Bushmaker T. Fischer R. (2018). SARS-Like Coronavirus WIV1-CoV Does Not Replicate in Egyptian Fruit Bats (Rousettus Aegyptiacus). Viruses 10 (12), 727. 10.3390/v10120727 PMC6316779 30572566

[B100] Viljoen M. Merwe M. v. d. Bower G. C. Levay P. Grobler A. (1997). Peripheral Blood Characteristics of Gravid Schreibers' Long-Fingered Bats, Miniopterus Schreibersii Natalensis (Microchiroptera: Vespertilionidae). South Afr. J. Sci. 93, 414–418.

[B101] Voigt C. C. Fritze M. Lindecke O. Costantini D. Pētersons G. Czirják G. Á. (2020). The Immune Response of Bats Differs between Pre-migration and Migration Seasons. Sci. Rep. 10 (1), 17384. 10.1038/s41598-020-74473-3 33060711 PMC7562910

[B102] Wang L.-F. Anderson D. E. (2019). Viruses in Bats and Potential Spillover to Animals and Humans. Curr. Opin. Virol. 34, 79–89. 10.1016/j.coviro.2018.12.007 30665189 PMC7102861

[B103] Washington I. M. Van Hoosier G. (2012). Clinical Biochemistry and Hematology. Labor. Rabbit, Guin. Pig, Hamster, Other Rodents, 57–116. 10.1016/B978-0-12-380920-9.00003-1

[B104] Weigl R. Jones M. L. Natur-Museum und Forschungs-Institut Senckenberg (2005). Longevity of Mammals in Captivity : From the Living Collections of the World : a List of Mammalian Longevity in Captivity. Stuttgart: E. Schweizerbart'sche.

[B105] Welch W. D. Miller R. D. (1984). Effect of Enflurane, Isoflurane, and Nitrous Oxide on the Microbicidal Activity of Human Polymorphonuclear Leukocytes. Anesthesiology 61 (2), 188–192. 10.1097/00000542-198408000-00012 6380345

[B106] Westhuizen J. v. d. (1978). The Diurnal Cycle of Some Energy Substrates in the Fruit Bat Rousettus Aegyptiacus. South Afr. J. Sci. 74 (3), 99. 10.10520/AJA00382353_4859

[B107] Widmaier E. P. Kunz T. H. (1993). Basal, Diurnal, and Stress-Induced Levels of Glucose and Glucocorticoids in Captive Bats. J. Exp. Zool. 265 (5), 533–540. 10.1002/jez.1402650509 8468542

[B108] Wightman J. Roberts J. Chaffey G. Agar N. S. (1987). Erythrocyte Biochemistry of the Grey-Headed Fruit Bat (Pteropus Poliocephalus). Comp. Biochem. Physiol. Part B Comp. Biochem. 88 (1), 305–307. 10.1016/0305-0491(87)90119-2 3677608

[B109] Wilkinson G. S. South J. M. (2002). Life History, Ecology and Longevity in Bats. Aging Cell 1 (2), 124–131. 10.1046/j.1474-9728.2002.00020.x 12882342

[B110] Wilson D. E. Mittermeier R. A. Martingell H. E. Leslie D. Jr. Olivé M. Elliott A. (2019). Handbook of the Mammals of the World. Barcelona: Lynx Edicions.

[B111] Wołk E. Bogdanowicz W. (1987). Hematology of the Hibernating Bat: Myotis Daubentoni. Comp. Biochem. Physiol. Part A Physiol. 88 (4), 637–639. 10.1016/0300-9629(87)90675-x 2892639

[B112] Xie J. Li Y. Shen X. Goh G. Zhu Y. Cui J. (2018). Dampened STING-Dependent Interferon Activation in Bats. Cell Host Microbe 23 (3), 297–301.e4. 10.1016/j.chom.2018.01.006 29478775 PMC7104992

[B113] Zhou P. Yang X.-L. Wang X.-G. Hu B. Zhang L. Zhang W. (2020). A Pneumonia Outbreak Associated with a New Coronavirus of Probable Bat Origin. Nature 579 (7798), 270–273. 10.1038/s41586-020-2012-7 32015507 PMC7095418

