# Supplementary material for: Baseline of Physiological Body Temperature and Hematological Parameters in Captive Rousettus aegyptiacus and Eidolon helvum Fruit Bats
Source: Front Physiol. 2022 Aug 29;13:910157. doi: 10.3389/fphys.2022.910157 (PMC9465388; doi:10.3389/fphys.2022.910157)
Supplement: Supplementary file 1 [file Table1.DOCX]

Supplementary Material

**Baseline of Physiological Body Temperature and Hematological Parameters in Captive *Rousettus aegyptiacus* and *Eidolon helvum* Fruit Bats**

Melanie Rissmann^1,2^, Virginia Friedrichs^3^, Nils Kley^1^, Martin Straube^4^, Balal Sadeghi^1^, Anne Balkema-Buschmann^1#^

**Supplementary Table 1:| Clinical chemistry values determined for *R. aegyptiacus* and *E. helvum* with statistical analysis of inter-species variation**

| **Parameter** | **Species** | **Mean** | **Min** | **Max** | **SEM** | ***P-value*** |
| --- | --- | --- | --- | --- | --- | --- |
| ALT  (U/l) | *R. aegyptiacus* | 48.20 | 26.00 | 90.00 | 2.07 | < 0.001*** |
|  | *E. helvum* | 26.40 | 17.00 | 43.00 | 1.21 |  |
| BUN  (mmol/l) | *R. aegyptiacus* | 2.97 | 0.90 | 4.60 | 0.13 | < 0.001*** |
|  | *E. helvum* | 1.62 | 1.00 | 3.30 | 0.10 |  |
| TP  (g/l) | *R. aegyptiacus* | 59.52 | 49.00 | 67.00 | 0.72 | < 0.001*** |
|  | *E. helvum* | 71.06 | 58.00 | 83.00 | 1.19 |  |
| ALP  (U/l) | *R. aegyptiacus* | 381.5 | 157.00 | 703.00 | 185.00 | n.a. |
|  | *E. helvum* | n.a. | n.a. | n.a. | n.a. |  |
| CREA  (µmol/l) | *R. aegyptiacus* | 47.53 | 18.00 | 70.40 | 2.36 | < 0.001*** |
|  | *E. helvum* | 29.23 | 18.00 | 52.00 | 2.06 |  |
| GLU  (mmol/l) | *R. aegyptiacus* | 3.45 | 0.60 | 10.00 | 0.48 | < 0.05* |
|  | *E. helvum* | 5.43 | 1.00 | 12.50 | 0.47 |  |

ALP = alkaline phosphatase; ALT = alanin-aminotransferase; BUN = blood urea nitrogen; CREA = creatinine; TP = total protein; GLU = glucose. Statistically significant differences were determined for clinical blood chemistry parameters (ALT, BUN, TP, ALP, CREA, GLU) between both species (⁎ = significant: *P*-value < 0.05; ⁎⁎ = very significant: *P*-value < 0.01; ⁎⁎⁎ = extremely or highly significant: *P*-value < 0.001).

**Supplementary Table 2: Hematology values determined for *R. aegyptiacus* and *E. helvum* with statistical analysis of inter-species variation**

| **Parameter** | **Species** | **Mean** | **Min** | **Max** | **SEM** | ***P-value*** |
| --- | --- | --- | --- | --- | --- | --- |
| HCT  (%) | *R. aegyptiacus* | 44.67 | 41.66 | 48.08 | 0.31 | 0.13 |
|  | *E. helvum* | 43.83 | 39.81 | 48.46 | 0.48 |  |
| HGB  (g/dl) | *R. aegyptiacus* | 17.36 | 15.60 | 19.20 | 0.15 | < 0.001*** |
|  | *E. helvum* | 16.38 | 14.80 | 17.90 | 0.20 |  |
| LY  (10^9^/l) | *R. aegyptiacus* | 12.51 | 4.97 | 24.59 | 0.96 | 0.33 |
|  | *E. helvum* | 14.20 | 6.84 | 33.70 | 1.55 |  |
| NE  (10^9^/l) | *R. aegyptiacus* | 1.00 | 0.08 | 3.62 | 0.20 | < 0.05* |
|  | *E. helvum* | 1.75 | 0.35 | 11.07 | 0.53 |  |
| RBC  (10^12^/l) | *R. aegyptiacus* | 12.90 | 11.55 | 14.49 | 0.14 | < 0.001*** |
|  | *E. helvum* | 10.3 | 0.05 | 11.20 | 0.53 |  |
| WBC  (10^9^/l) | *R. aegyptiacus* | 13.71 | 5.47 | 26.11 | 0.97 | 0.14 |
|  | *E. helvum* | 16.55 | 7.85 | 37.46 | 1.83 |  |

HCT = hematocrit; HGB = hemoglobin; LY = lymphocytes; NE = neutrophil granulocytes; WBC = white blood cells; RBC = red blood cells. Variations determined for the parameters HGB, NE and RBC were statistically significant between both species (⁎ = significant: *P*-value < 0.05; ⁎⁎ = very significant: *P*-value < 0.01; ⁎⁎⁎ = extremely or highly significant: *P*-value < 0.001). There was no statistically significant difference in HCT, LY and WBC between both species (*P*-value > 0.05).

**Supplementary Table 3: Hematology values determined for *R. aegyptiacus* and *E. helvum* that were excluded from further statistical analysis**

| **Parameter** | **Species** | **Mean** | **Min** | **Max** |
| --- | --- | --- | --- | --- |
| BA  (10^9^/l) | *R. aegyptiacus* | 0.001 | 0 | 0.10 |
|  | *E. helvum* | 0.01 | 0 | 0.10 |
| EO  (10^9^/l) | *R. aegyptiacus* | 0.01 | 0 | 0.01 |
|  | *E. helvum* | 0.01 | 0 | 0.02 |
| MON  (10^9^/l) | *R. aegyptiacus* | 0.17 | 0.03 | 0.85 |
|  | *E. helvum* | 0.21 | 0.04 | 0.74 |
| MCH  (pg) | *R. aegyptiacus* | 13.47 | 12.40 | 14.40 |
|  | *E. helvum* | 14.87 | 0 | 16.40 |
| MCHC  (g/dl) | *R. aegyptiacus* | 38.85 | 36.90 | 40.40 |
|  | *E. helvum* | 35.58 | 0 | 40.20 |
| MCV  (fl) | *R. aegyptiacus* | 34.60 | 31.00 | 38.00 |
|  | *E. helvum* | 41.90 | 39.00 | 45.00 |
| PLT  (10^9^/l) | *R. aegyptiacus* | 586.23 | 205.00 | 1313.00 |
|  | *E. helvum* | 844.15 | 25.00 | 1736.00 |
| PCT  (%) | *R. aegyptiacus* | 0.325 | 0.10 | 0.72 |
|  | *E. helvum* | 0.56 | 0.02 | 1.24 |
| MPV  (fl) | *R. aegyptiacus* | 5.48 | 5.00 | 6.40 |
|  | *E. helvum* | 6.67 | 5.60 | 7.80 |
| PDW  (fl) | *R. aegyptiacus* | 7.00 | 6.00 | 9.00 |
|  | *E. helvum* | 9.04 | 6.00 | 14.50 |
| RDW  (fl) | *R. aegyptiacus* | 26.86 | 25,80 | 28.90 |
|  | *E. helvum* | 29.26 | 18.00 | 32.80 |

BA = basophil granulocytes; EO = eosinophil granulocytes; MON = monocytes; MCH = mean corpuscular hemoglobin; MCHC = mean corpuscular hemoglobin concentration; MCV = mean corpuscular volume; PLT = platelet count; PCT = plateletcrit; MPV = mean platelet volume; PDW = platelet distribution width; RDW = red cell distribution.
